# Supplementary material for: “Pour some sugar on me”—Environmental Candida albicans isolates and the evolution of increased pathogenicity and antifungal resistance through sugar adaptation
Source: PLoS Pathog. 2025 Oct 9;21(10):e1013542. doi: 10.1371/journal.ppat.1013542 (PMC12510538; doi:10.1371/journal.ppat.1013542)
Supplement: S1 Table — (DOCX) [file ppat.1013542.s001.docx]

| **Name** | **Description** | **Source** |
| --- | --- | --- |
| SC5314 | Reference strain | [91] |
| Oak 1 | Isolate from the bark of old oak trees, original name: NCYC4144 | [17, 20] |
| Oak 2 | Isolate from the bark of old oak trees, original name: NCYC4145 | [17, 20] |
| Oak 3 | Isolate from the bark of old oak trees, original name: NCYC4146 | [17, 20] |
| Evo | Galactose-adapted Oak 1 strain, evolution was performed for 6 weeks | This study |

**S1 Table. Strains used in this study.**
