# Supplementary material for: “Pour some sugar on me”—Environmental Candida albicans isolates and the evolution of increased pathogenicity and antifungal resistance through sugar adaptation
Source: PLoS Pathog. 2025 Oct 9;21(10):e1013542. doi: 10.1371/journal.ppat.1013542 (PMC12510538; doi:10.1371/journal.ppat.1013542)
Supplement: S2 Table — (DOCX) [file ppat.1013542.s002.docx]

| **Name** | **Sequence 5’ to 3’** | **Gene** |
| --- | --- | --- |
| ECE1 fw | CACTGGTGTTCAACAATCCAT | *ECE1* |
| ECE1 rev | AGCATTTTCAATACCGACAG | *ECE1* |
| ACT1 fw | TCAGACCAGCTGATTTAGGTTTG | *ACT1* |
| ACT1 rev | GTGAACAATGGATGGACCAG | *ACT1* |
| EFB1 fw | ATTGAACGAATTCTTGGCTGAC | *EFB1* |
| EFB1 rev | CATCTTCTTCAACAGCAGCTTG | *EFB1* |

**S2 Table. Primers used for RT-qPCR.**
