# Supplementary figures and images for: “Pour some sugar on me”—Environmental Candida albicans isolates and the evolution of increased pathogenicity and antifungal resistance through sugar adaptation
Source: PLoS Pathog. 2025 Oct 9;21(10):e1013542. doi: 10.1371/journal.ppat.1013542 (PMC12510538; doi:10.1371/journal.ppat.1013542)

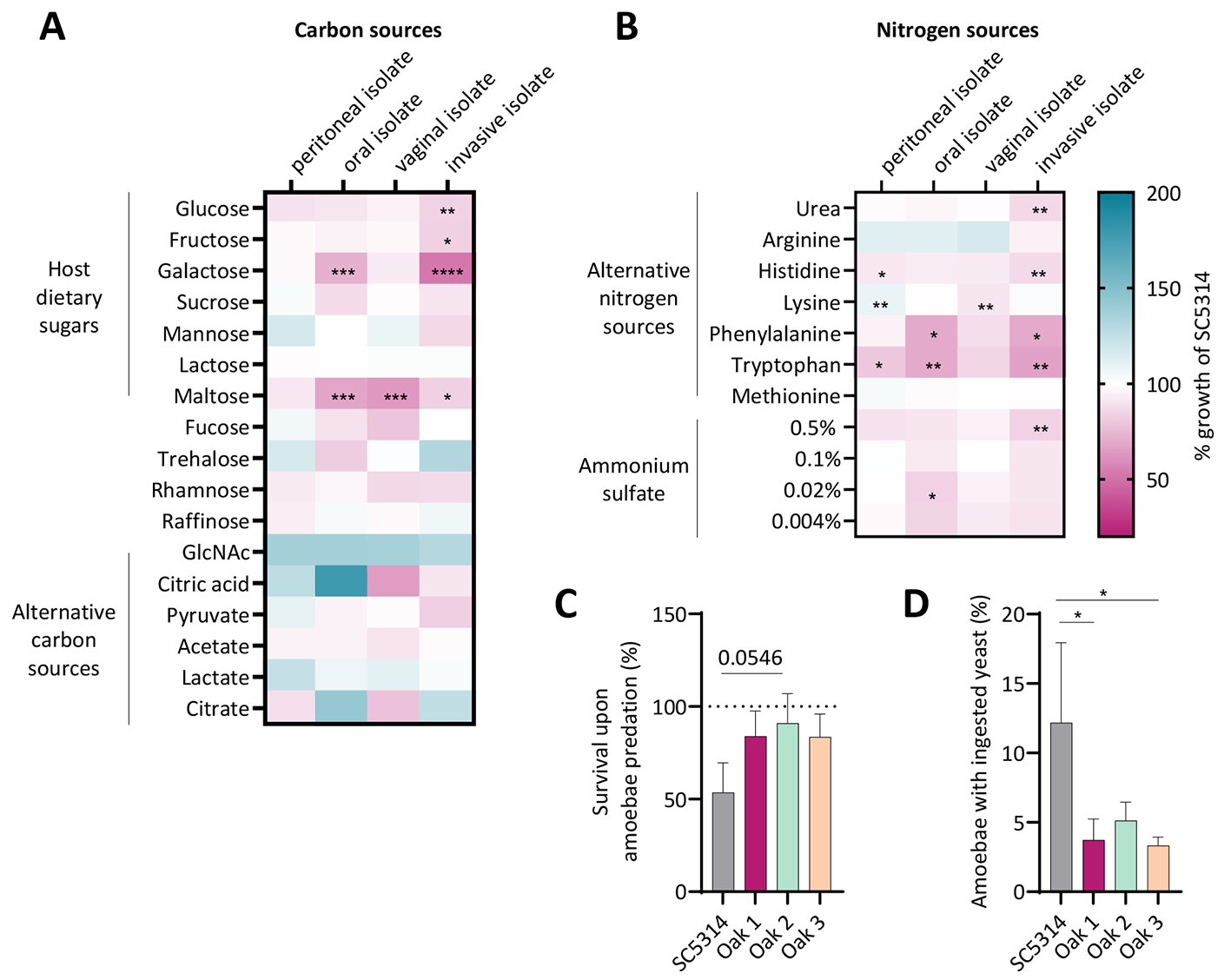

Supplement: S1 Fig — Growth was measured as growth curves at 30 °C in different media comprising specific carbon (A) or nitrogen (B) sources. The growth is shown as area under the curve relative to the reference strain SC5314 (% growth). Asterisks indicate significance compared to SC5314 calculated using a one-way ANOVA with Dunnett’s multiple comparisons test (* p < 0.05, ** p < 0.01, *** p < 0.001, **** p < 0.0001) (n = 3). (C) Survival of C. albicans yeast cells after 3 h of co-incubation with P. aurantium. Results were compared using a one-way ANOVA with Tukey’s multiple comparisons test (n = 3). (D) Percentage of P. aurantium cells containing internalized C. albicans yeast cells after 2 h. Results were compared using a one-way ANOVA with Tukey’s multiple comparisons test (* p < 0.05) (n = 3). (TIF) [file ppat.1013542.s004.tif]

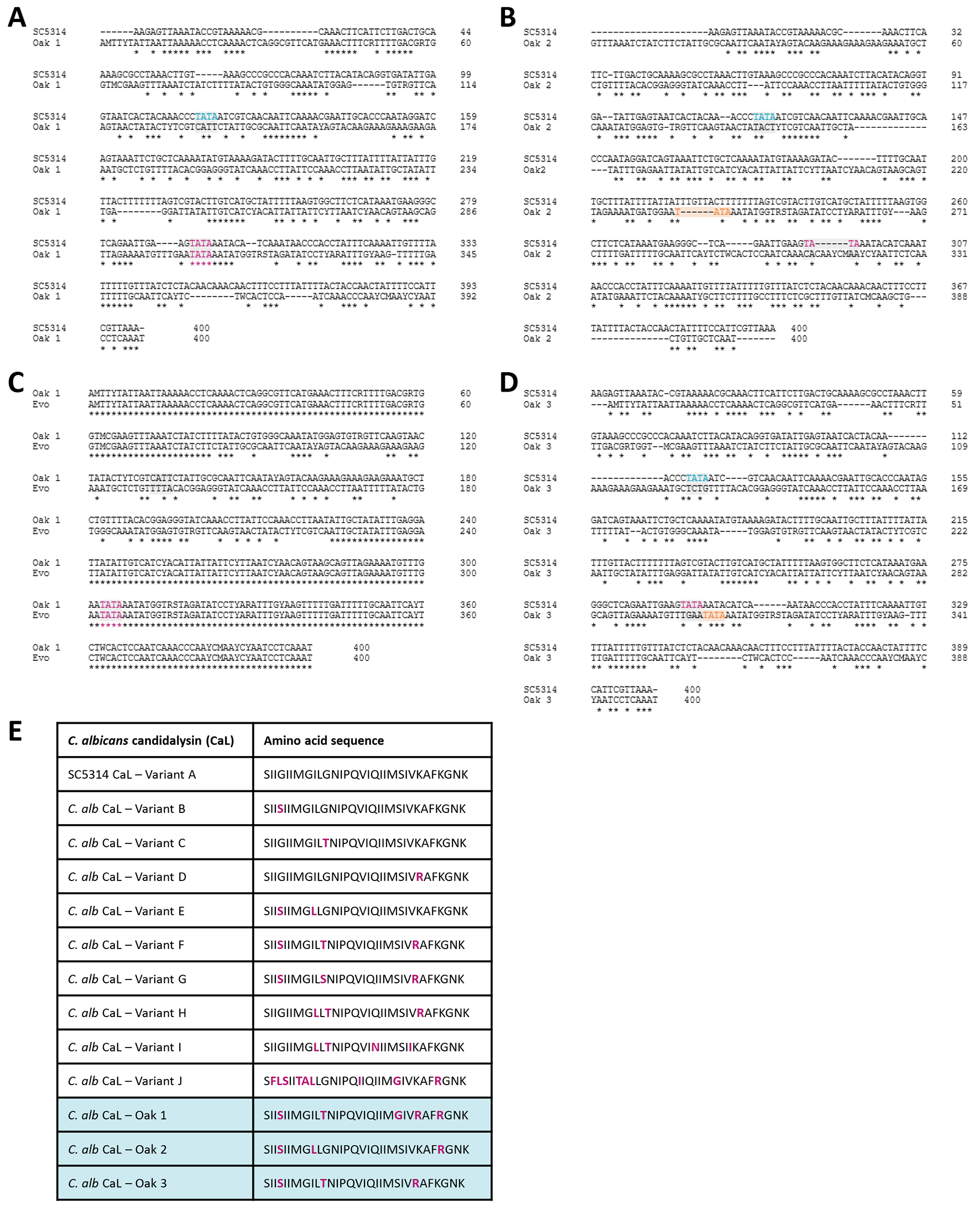

Supplement: S2 Fig — (A-D) The promoter region is shown 400 bp upstream of ECE1. Pink highlights the first TATA box and blue the second, less important TATA box as observed in [47]. Orange indicates a newly identified TATA box in the sequence. Alignments were made using Clustal Omega. Identical amino acids are indicated as asterisks (*). (E) Candidalysin sequences from oak tree isolates compared to clinical isolate sequences (taken from [46]). Different amino acids are indicated in purple. Oak 1 and Oak 2 have unique candidalysin sequences, Oak 3 has the same sequence as variant F, previously found in the clinical strains. (TIF) [file ppat.1013542.s005.tif]

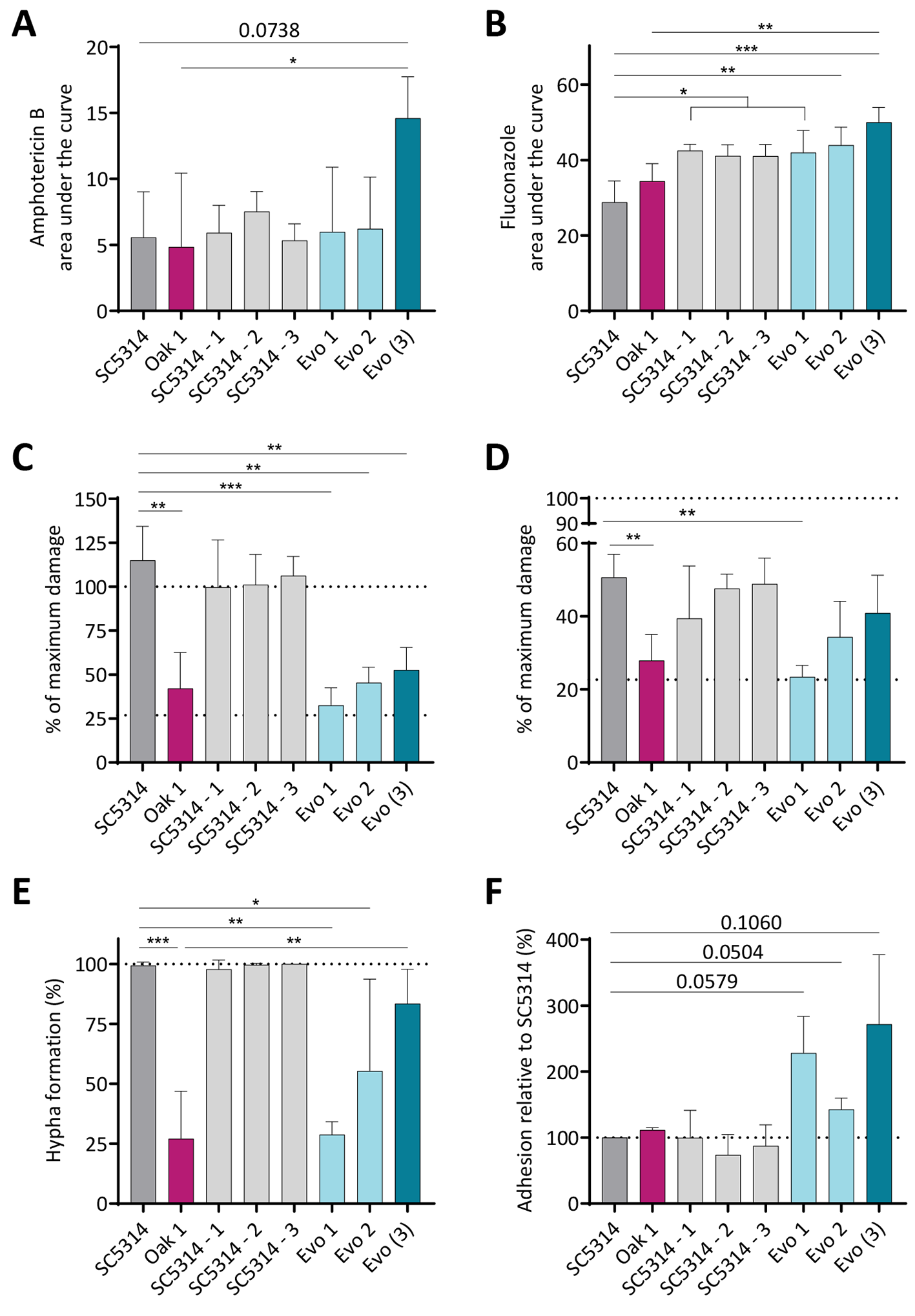

Supplement: S3 Fig — The strains Evo 1, Evo 2, and Evo 3 are the triplicates kept during the sugar micro-evolution experiment and are all derived from Oak 1. The strain further characterized in this study is Evo 3, and was named ‘Evo’ to simplify the nomenclature. (A, B) Antifungal resistance of the galactose-evolved strains was assessed by performing growth curves with YPD supplemented with either 32 µg/ml fluconazole or 0.5 µg/ml amphotericin B at 30 °C for 72 h, and is depicted as area under the curve (n = 3). (C) Damage to oral cells and (D) intestinal cells was evaluated by measuring the LDH release 48 h post infection (oral n = 3, intestinal n = 4). The lower dashed line indicates the uninfected control. (E) Hypha formation was determined by microscopic evaluation counting the number of hypha-forming cells relative to all present C. albicans cells on oral cells 3 h post infection (n = 3). (F) Adhesion of the C. albicans strains was assessed by fixing and staining the fungal cells 1 h post infecting oral cells. The average number of attached C. albicans fungal cells per microscopic image is plotted relative to the reference strain SC5314 (n = 3). (A-F) For all graphs, significances were calculated by using a one-way ANOVA with Tukey’s multiple comparisons test (* p < 0.05, ** p < 0.01, *** p < 0.001). Data of all graphs are depicted partially in the main Figs 5 and 7. For panel (C), (D), and (F), the difference between Oak 1 and Evo was not statistically significant. (TIF) [file ppat.1013542.s006.tif]

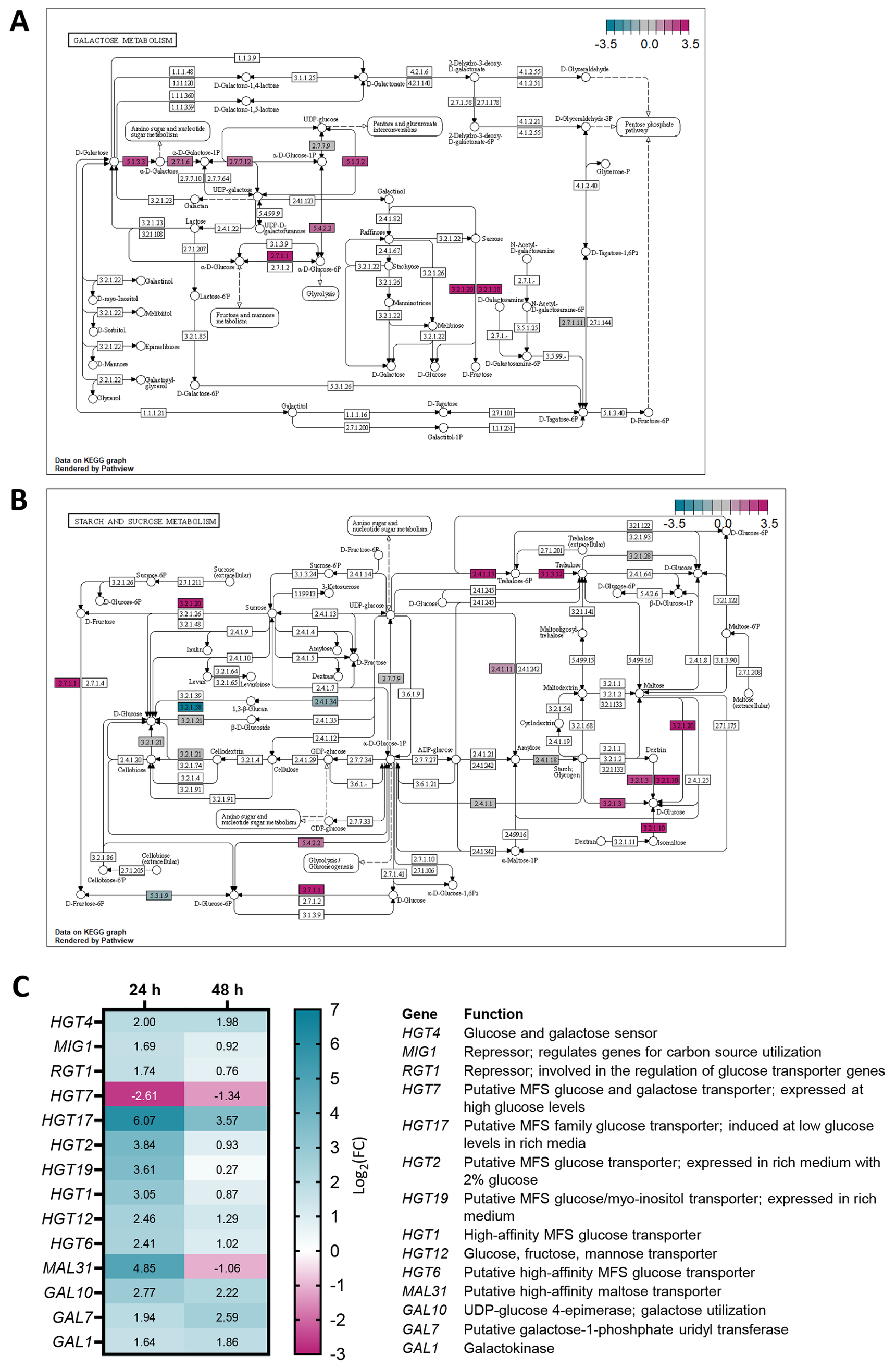

Supplement: S4 Fig — (A, B) Galactose metabolism (A) as well as starch and sucrose metabolism KEGG pathways (B) of the sugar-evolved strain relative to its parental oak tree isolate 1. Gene expression was assessed 24 h p.i. on intestinal epithelial cells. Fold change is shown with the indicated color scale. Genes which are depicted in grey were not included in the analysis. (C) Log2(fold change) of genes involved in glucose and galactose sensing, transport, as well as in the downstream signaling pathways [112] of the sugar-adapted strain relative to its parental strain (oak tree isolate 1) on intestinal epithelial cells. Gene functions were taken from candidagenome.org. (TIF) [file ppat.1013542.s007.tif]

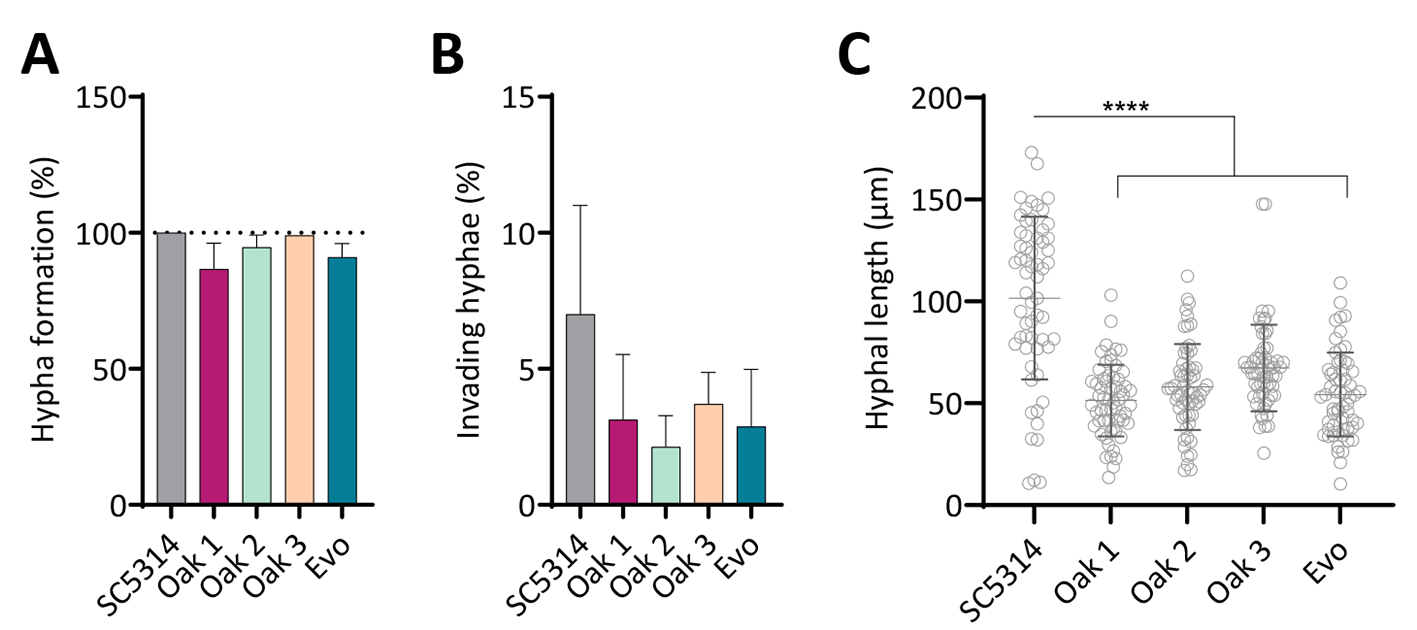

Supplement: S5 Fig — (A) Hypha formation was determined by microscopic evaluation counting the number of hypha-forming cells relative to all present C. albicans cells 6 h post infection (n = 3). (B) Hyphal length was measured after 6 h after infecting intestinal cells (n = 3). (C) Invasion was measured by infecting intestinal cells, fixing, and differentially staining 6 h post infection. Invasion was calculated as percentage of invading hyphae compared to all counted fungal cells. Statistical significance was calculated using a one-way ANOVA with Tukey’s multiple comparisons test (**** p < 0.0001) (n = 3). The difference between Oak 1 and Evo was not statistically significant. (TIF) [file ppat.1013542.s008.tif]

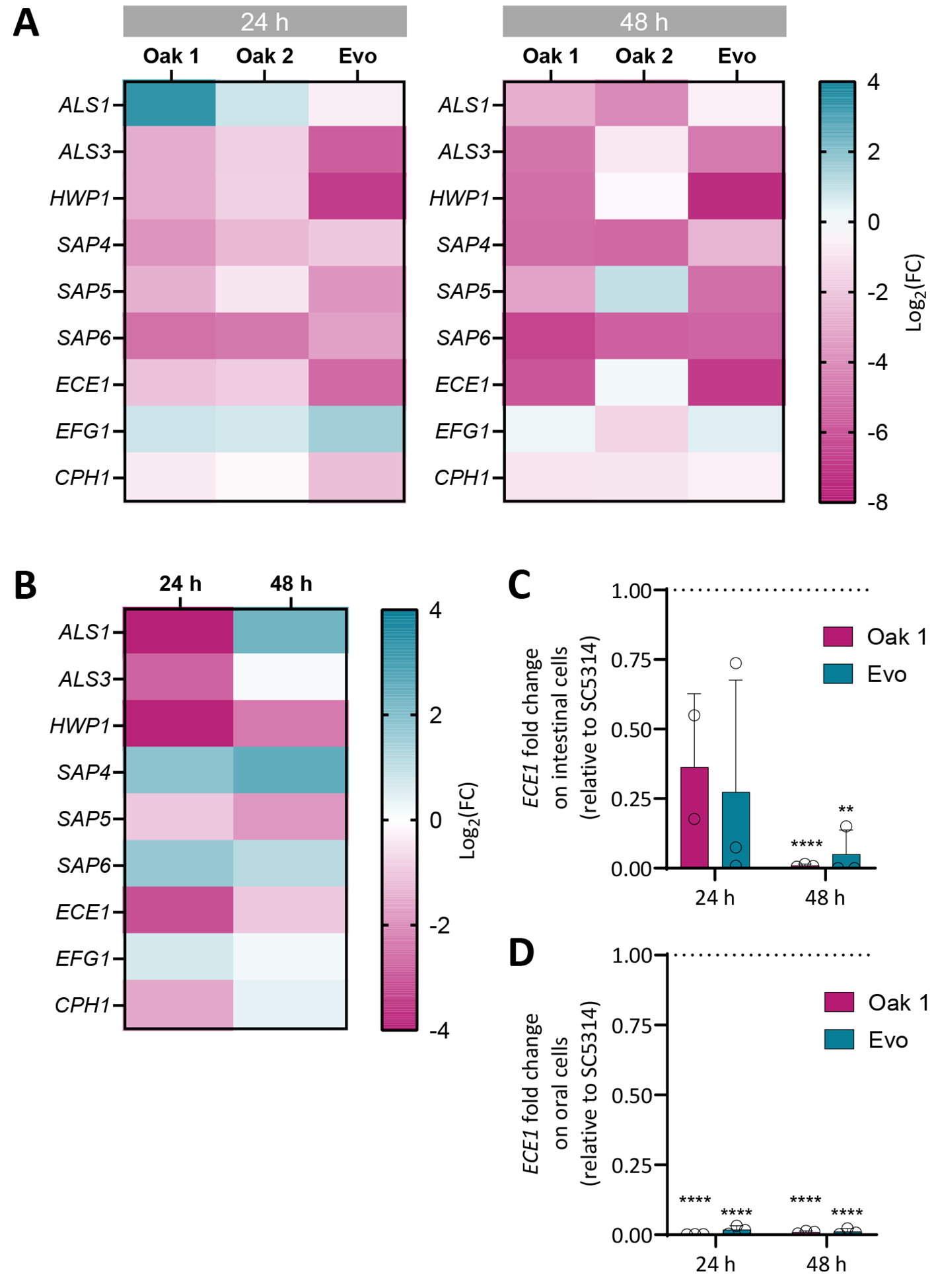

Supplement: S6 Fig — (A) Log2(fold change) of some hypha-associated genes for the non-virulent oak tree isolate 1, the highly virulent oak tree isolate 2, and the sugar-adapted strain (Evo) relative to SC5314 24 h or 48 h post infection of intestinal epithelial cells. (B) Log2(fold change) of some hypha-associated genes of the sugar-adapted strain relative to its parental strain (oak tree isolate 1). For both (A, B), expression was assessed via RNA sequencing. (C, D) ECE1 expression of the sugar-adapted strain on intestinal (C) and oral epithelial (D) cells. Expression was assessed via qPCR and is shown as fold change relative to SC5314 at the indicated time points. The expression is normalized to ACT1. Statistical significance was calculated using a one-sample t-test comparing the mean of the samples with a hypothetical mean of 1 (** p < 0.01, **** p < 0.0001) (n = 3). The difference of Oak 1 and Evo was not statistically significant. (TIF) [file ppat.1013542.s009.tif]

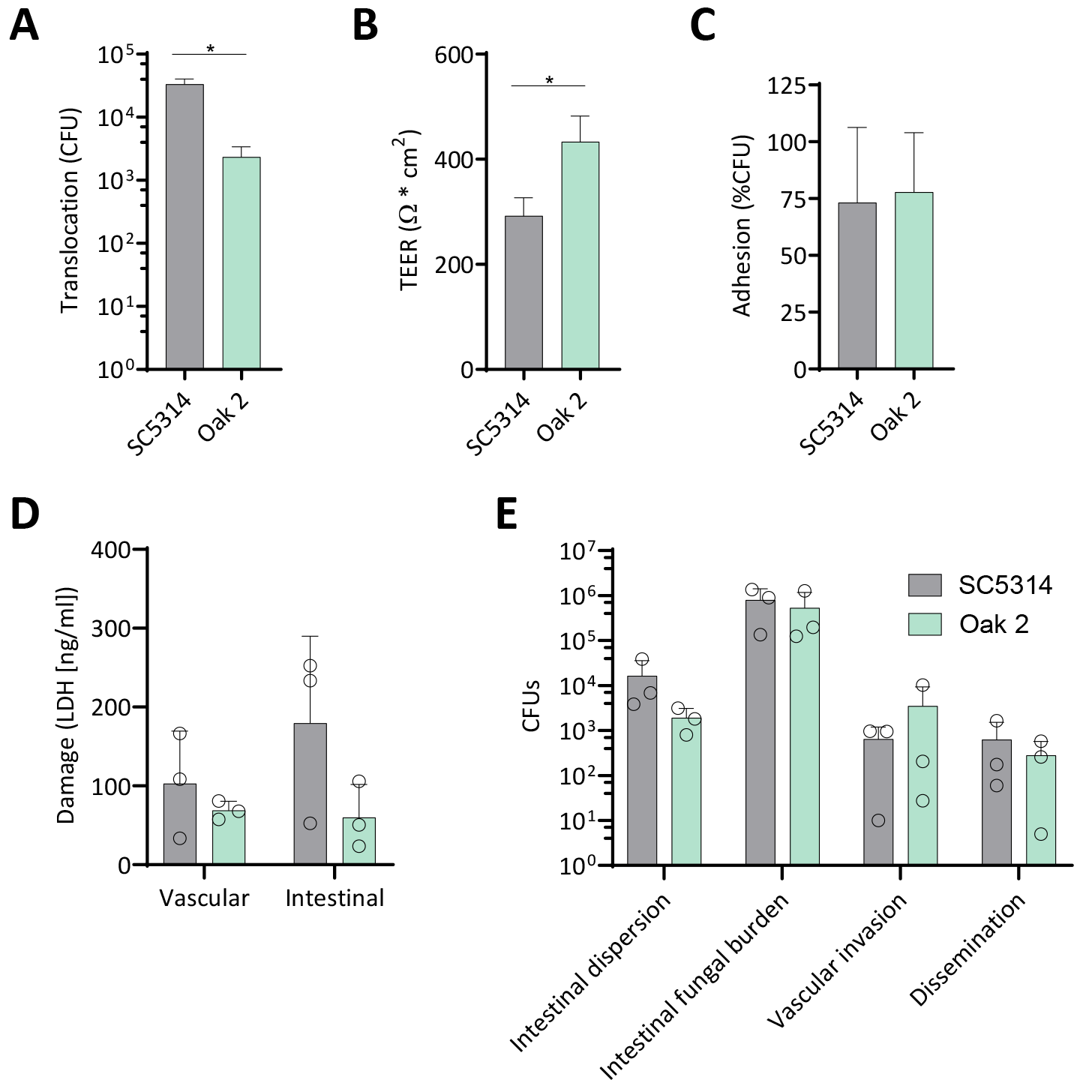

Supplement: S7 Fig — (A) Translocation of the strains was measured in an intestinal transwell model by plating the translocated colonies 24 h post infection. Significance was calculated using a Welch’s t-test (* p < 0.05) (n = 3). (B) Barrier integrity was determined by measuring TEER (transepithelial electrical resistance) in an intestinal transwell model 24 h after infection. Significance was calculated using a Welch’s t-test (* p < 0.05) (n = 3). (C) Percentage of adhesion to the gut epithelium in the gut-on-chip model was determined by plating the amount of non-attached C. albicans in the flow-through relative to the injected inoculum (n = 3). (D) Damage was measured as LDH release from both, the gut side and the vascular compartment of the gut-on-chip 24 h post infection (n = 3). (E) Fungal burden was determined by plating the intestinal dispersion (gut flow-through), intestinal fungal burden (intestinal lysate), vascular invasion (vascular lysate), and dissemination (vascular flow-through) 24 h post infection (n = 3). To show the variation between the different gut-on-chip experiments, the individual values of the biological replicates are shown in (D) and (E). (TIF) [file ppat.1013542.s010.tif]

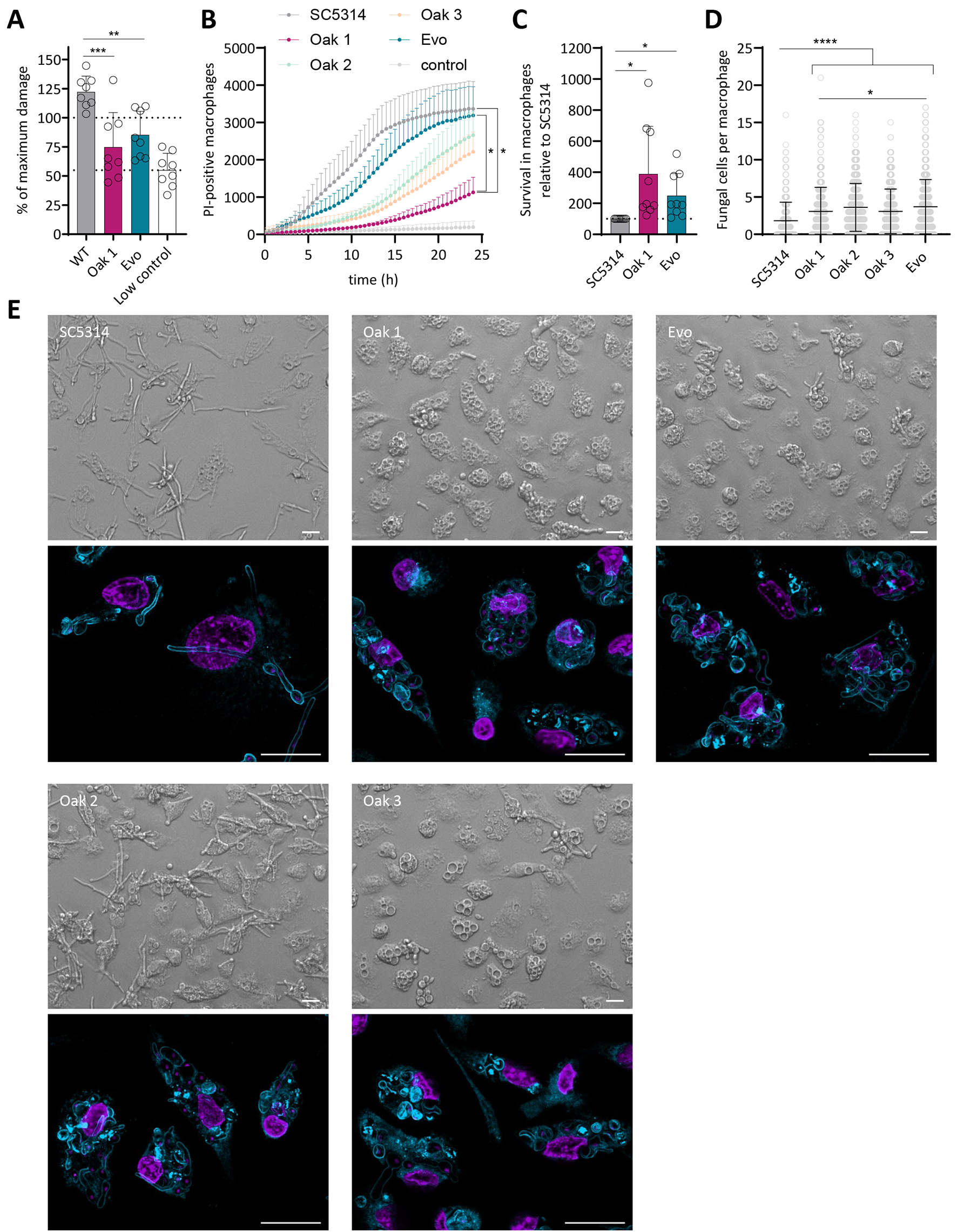

Supplement: S8 Fig — (A) Damage to monocyte-derived macrophages was determined after 24 h by measuring LDH release. Each dot represents one donor. Significance was calculated by using a one-way ANOVA with Tukey’s multiple comparisons test (** p < 0.01, *** p < 0.001) (8 donors). The difference of Oak 1 and Evo was not statistically significant. The lower dashed line indicates the uninfected control. (B) Host cell lysis of monocyte-derived macrophages was measured by propidium iodide staining over the course of 24 h. Significance was calculated using a two-way ANOVA with repeated measures and Tukey’s multiple comparison test (* p < 0.05) (4 donors). (C) Intracellular survival in human monocyte-derived macrophages of the C. albicans strains was assessed 3 h post infection by lysing and plating the intracellular fungal cells. Survival is depicted relative to the reference strain SC5314. Each dot represents one donor. Significance was calculated by using a one-sample t-test comparing the mean of the samples with 100% (* p < 0.05) (9 donors). The difference of Oak 1 and Evo was not statistically significant. (D) Fungal cells per macrophage were determined 3 h post infection by staining fungal cells (ConA-AlexaFluor647, blue) and cell nuclei (DAPI, purple). Images were taken at 63 × magnification with immersion oil (scale bar, 20 µm), and fungal cells per macrophage were counted. Each dot represents one macrophage. Infected macrophages from 4-6 donors were counted. Significance was calculated by using a one-way ANOVA with Tukey’s multiple comparisons test (* p < 0.05, **** p < 0.0001). (E) Phase contrast microscopic pictures were taken 3 h post infecting human monocyte-derived macrophage at 10x magnification, and microscopy images were taken from quantification in (C) at 63 × magnification with immersion oil (scale bar, 20 µm). Samples were stained with DAPI (nuclei staining, purple) and Concanavalin A-AlexaFluor647 (fungal staining, blue). The pictures for SC5314, Oak 1, Oak 2, and Oak 3 are [file ppat.1013542.s011.tif]

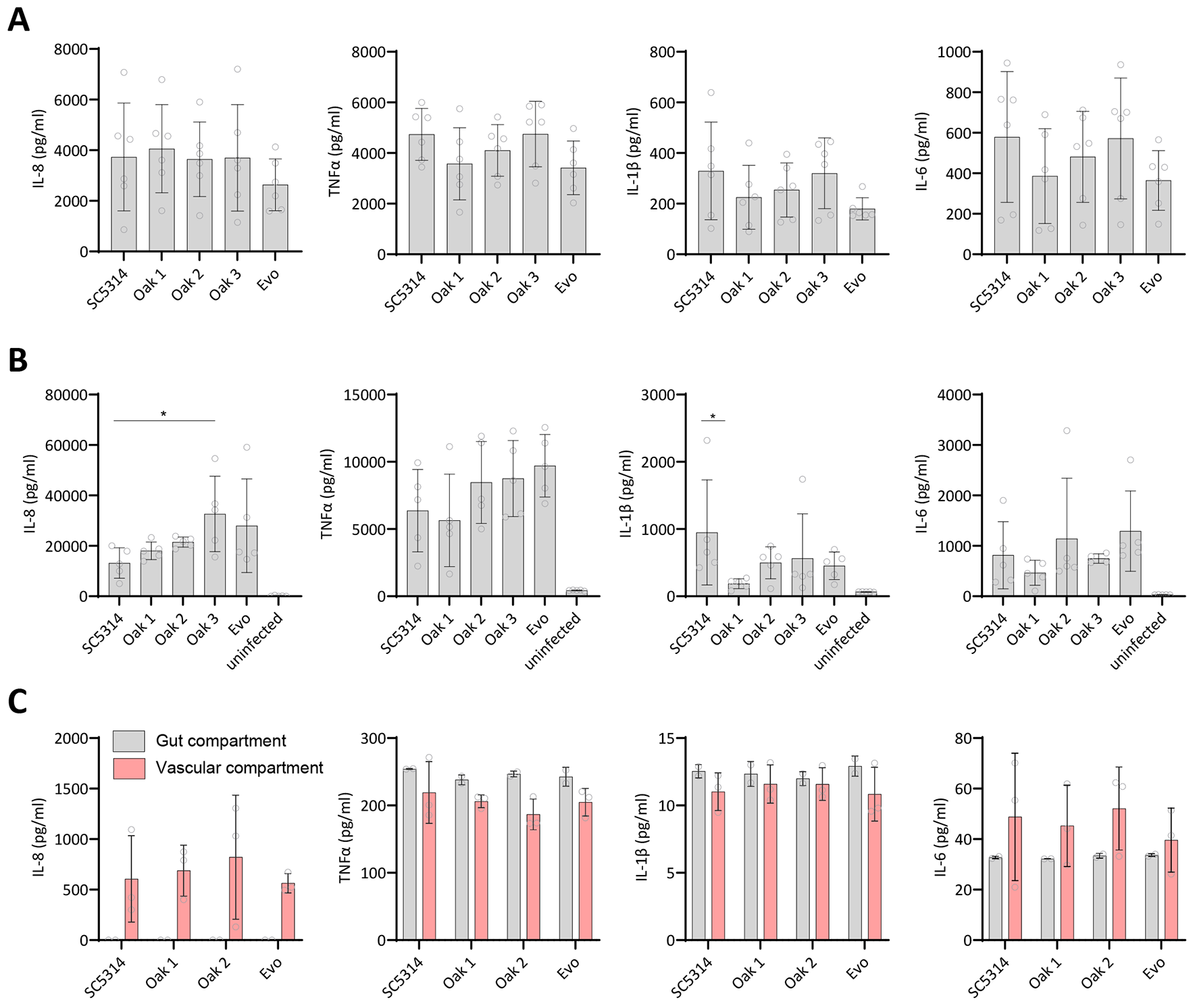

Supplement: S9 Fig — Release of IL-8, TNF-α, IL-1β, and IL-6 was determined in supernatants of (A) infected whole blood after 4 h of infection (6 donors), (B) by infected primary human macrophages 24 h post infection (5 donors), and (C) in supernatants of the vascular and gut compartment of the organ-on-chip model 24 h post infection (n = 3). Significance in (B) was calculated by using a one-way ANOVA with Dunnett’s multiple comparisons test (* p < 0.05). The difference of Oak 1 and Evo was not statistically significant. (TIF) [file ppat.1013542.s012.tif]
