# Supplementary material for: “Pour some sugar on me”—Environmental Candida albicans isolates and the evolution of increased pathogenicity and antifungal resistance through sugar adaptation
Source: PLoS Pathog. 2025 Oct 9;21(10):e1013542. doi: 10.1371/journal.ppat.1013542 (PMC12510538; doi:10.1371/journal.ppat.1013542)
Supplement: Attachment — Submitted filename: Cover Letter and Response to Reviewers.pdf [file ppat.1013542.s013.pdf]

Leibniz Institute
for Natural Product Research
and Infection Biology
Hans Knöll Institute

Leibniz-HKI · Adolf-Reichwein-Strasse 23 · 07745 Jena · Germany

To
Editors
PLOS Pathogens

Jena, 30 July 2025

Prof. Bernhard Hube
Dept. of Microbial
Pathogenicity Mechanisms

+49 3641 532-1401
bernhard.hube@leibniz-hki.de

Leibniz Institute for Natural Product Research and Infection Biology
Hans Knöll Institute

Visitors' address
Beutenbergstrasse 11a
07745 Jena

Mailing and delivery address
Adolf-Reichwein-Strasse 23
07745 Jena

Amtsgericht Jena VR 230298

Board of Directors
Prof. Dr. Axel A. Brakhage
Elke Jäcksch

Bank Accounts
Commerzbank Jena
DE98 8204 0000 0258 5370 00
BIC COBADEFFXXX

Sparkasse Jena
DE72 8305 3030 0000 0006 71
BIC HELADEF1JEN

VAT-ID DE153925472
Tax number 162/141/08244

www.leibniz-hki.de

Re: Revision of PPATHOGENS-D-25-00859

Dear editors of *PLOS Pathogens*,

Thank you for your e-mail from the 2nd of June, in which you expressed interest in our manuscript pending a revision of the points raised by you and the reviewers.

We have now addressed all the points that were raised during this peer review. Specifically, we now better show the likely environmental source of the strains and how they in fact differ from many known *C. albicans* strains. We also provide data from new experiments to better address some of the mechanisms, as requested by some reviewers. Finally, we have made extensive text changes where the reviewer comments made clear that our text was clear as we had hoped.

A detailed response to all the helpful comments by you and the reviewers can be found on the next pages. We are confident that these revisions significantly improved our manuscript, and we hope that you now find it ready for publication in *PLOS Pathogens*.

We look forward to your response.

Sincerely,

Prof. Bernhard Hube

Leibniz Association

Point-by-point response to points raised by the reviewers

Additional Editor Comments:
The reviewers thought the topic of your manuscript was interesting, but they all had major concerns. This will require extensive new experiments to address their comments if you choose to send in a revised manuscript.

Response:
To deal with all comments, we have performed a series of new experiments and added multiple new or expanded data sets including

(1) Growth curves of all clinical isolates included in the study
(2) Data on amoebae infections with the oak tree-derived isolates
(3) Live cell imaging with human monocyte-derived macrophages
(4) Microscopic images for primary human macrophage interactions with the fungi
(5) Survival assay data with primary human macrophages
(6) Infection data from an *ex vivo* whole blood model with Oak 3
(7) Candidalysin sequence alignment with sequences from clinical isolate

One major underlying concern of the reviewers, expressed most strongly by Reviewers 1 and 3, was that there is no clear evidence that the isolates were truly "environmental". For example, it can not be ruled out that they came from an animal host. This led to questions as to whether these new strains were really outliers relative to the range of known C. albicans clinical isolates, which negatively impacted the significance. I realize this is a difficult question to address, but new experiments will be required to address this point to gain stronger support from the reviewers.

Response:
This may have been a misunderstanding. We would argue that this is a question of definition of the term "environmental". In our view, a transient colonization of warm-blooded animals would still be "environmental" in contrast to a permanent and sole association with humans. Such a transient association with animals is also discussed for "soil" environmental pathogenic fungi such as *Cryptococcus neoformans*, *Histoplasma capsulatum* or *Coccidioides immitis* [1-4], and it still would comprise a significantly different life history of these strains compared to the typical, constantly human-associated isolates.
Furthermore, it seems that maybe not all reviewers are aware of the fact that our manuscript is based on the 2019 work of Bensasson *et al*. in Genetics [5], who isolated the Oak tree strains used in our study. They extensively discussed the original habitat of these isolates and compared the corresponding genome sequences with clinical isolates. To cite from this work: "Here, we generated genome sequences for three strains of *C. albicans* from oak bark from the New Forest in the United Kingdom (Robinson *et al.* 2016). These are the first *C. albicans* genomes sequenced from a nonanimal source, and we compared them to the genomes of over 200 strains from humans and animals (Muzzey *et al.* 2013; Hirakawa *et al.* 2015; Ropars *et al.* 2018). Although they were collected from the same woodland, these oak strain genomes are genetically diverged from one another, and are more similar to diverged clinical lineages than they are to each other. However, oak strains differ from clinical strains in showing higher levels of genome-wide heterozygosity, **suggesting that they were subject to different selection or mutation pressures.** The genetic diversity of *C. albicans* in this woodland implies that they have **lived there long enough for distinct lineages to accumulate** on unusually old trees. Therefore, *C. albicans* can live **in the oak environment for prolonged periods**." (emphasis ours).
Based on these previous investigations, we are rather confident that the strains can be considered "environmental", as in contrast to "human-associated". We have changed the text to better describe the environmental nature of the isolates and their origin by more extensively referring to the original publication in the revised manuscript (*e.g.* **line 421**, **line 490-496**).

Another weakness pointed out by the reviewers is that the manuscript was generally descriptive. Phenotypic differences were observed, but the underlying mechanisms were not identified. This was also a weakness for the evolution studies. These concerns will require significant new experiments to address.

Response:
We have addressed this criticism with several new experiments and better explanations where we think that our experiments already showed likely mechanisms. Overall, there remains a descriptive part in this manuscript, which we however do not consider a weakness, as part of our approach was to characterize, in detail, the

environmental strains in comparison to the classically used clinical strains. We followed up on several of the relevant phenotypes to find the underlying mechanism, but as in any scientific study, some questions remain even after our investigations. Please see the responses to the individual reviewer questions on how we think that together with our new data, we have addressed this concern.

The reviewers also had major concerns about the design and execution of the experiments. For example, all three reviewers had questions about the evolution studies. Reviewer 1 had questions about the several experiments, including the macrophage studies and the statistical analysis of the data. Reviewer 3 pointed out the lack of standard conditions for different experiments, which made it difficult to compare results. Addressing these concerns will also require new experiments.

Response:
We have dealt with all points raised by the reviewers and performed several new experiments and added new data as requested by all three reviewers.

Part I - Summary

Reviewer #1:
This manuscript compares three C. albicans isolates from oak trees in the UK to the lab type strain SC5314, a human bloodstream isolate, to assess phenotypic differences that may be relevant to environmental to human adaptation and infection. As expected these strains have substantial genomic and phenotypic differences relative to the lab strain. One of these strains is less fit in standard sugar-replete laboratory conditions and repeated passage in sugar-rich media resulted in one lineage with more pathogenic-like traits. This was associated with greater hyphal growth and cell damage.

In general this is an interesting study using rare environmental isolates and this paper assumes that the site of isolation makes them fundamentally different. This is not what the data shows: these strains are well within the phenotypic diversity that has long been known for C. albicans. Differences in host cell interactions and propensity for hyphal morphogenesis are well established amongst clinical and commensal strains; it does not appear that these oak tree-associated strains are unusual in their variation. If they were considered more in the context of the known phenotypic diversity, it would be a useful complement to the literature. One of the strains appears to represent a new genomic clade, but this is not a focus of this manuscript.

Response:
These are fair points and we fully agree with Reviewer #1. In fact, the underlying hypothesis at the beginning of our experiments was that the oak tree isolates should have a lower pathogenic potential than clinical isolates. However, the surprising outcome of our research was that there is very little difference to clinical isolates. Even more surprising to us was the fact that one strain induced damage to the same extent as known highly invasive clinical isolates – the strain that represents the new genomic clade. To us, this is one of the major points of our work: That despite their different origin (the support for which we now describe in more detail), the oak tree-derived strains can behave like fully human-adapted clinical *C. albicans* isolates in all tests of virulence potential. To address the points raised here by Reviewer #1, we now describe our original hypothesis in more detail and we have also introduced a new part into the discussion where we emphasize that the oak tree strains are indeed and unexpectedly within the variability range of known clinical *C. albicans* strains (**line 425-428 and 431-435**).

There is an assumption that these environmental isolates, which are quite rare relative to human isolates, represent a latent environmental reservoir of C. albicans that seeds human colonization. This is the primary basis for the significance of this manuscript, but there is simply no evidence to suggest this is true. Humans are not the only warm-blooded animals capable of harboring C. albicans and it is more likely that these isolates are deposited from animal contamination rather than populations of proliferating fungi on trees. The genomic diversity of these isolates from the same type of tree in the same national park is more consistent with bird droppings (for example) than some reservoir with which humans come in contact.

Response:
As discussed above, many of the underlying assumptions of our manuscript are based on Bensasson *et al*. [5], who isolated the Oak tree isolates used in our study. In their work, they extensively discussed the most likely original habitat of these isolates and analyzed the corresponding genome sequences in comparison with clinical isolates. Their data support the hypothesis of a continued existence of these strains on oak trees [5]. In addition,

recent publications have shown that *C. albicans* can be isolated more readily from environmental sources like berries or plant leaves than usually assumed [4-9]. As described above, we present these considerations and the original findings by Bensasson more clearly now in our own work (**line 421**, **line 487-496**).
That said, we fully agree that even the oak-associated strains could have been, at least transiently, in contact with warm-blooded animals. Indeed, *Candida albicans* has recently been isolated from bird droppings [4], which fully supports the point that the oak tree isolates could have been transiently associated with animals.
However, we would argue that this would still mean they represent environmental isolates in comparison to constantly human-associated commensals. Indeed, many "classical" environmental pathogenic fungi, such as *Histoplasma capsulatum, Coccidioides immitis* or *Cryptococcus neoformans*, can be readily isolated from pigeon droppings or rodents [1-4], but are still considered environmental "soil" fungi in the literature.
Most of this information can now be found in the revised paper as a dedicated new paragraph in the discussion (**line 490-496**).

The authors are right that there is much unknown about the acquisition and stability of Candida colonization in humans, but the evidence points to person-to-person transmission (e.g., mother to child) rather than environmental contacts and while some speculation otherwise is healthy, this goes a bit far.

Response:
We agree that person-to-person transmission is the most likely source for commensal *C. albicans* strains today. We do not suggest that the environmental strains are a major source of colonizing strains, but we propose that it is possible that early humans (*e.g.* with a hunter-gatherer lifestyle) have been first colonized with *C. albicans* strains form the environment. This process may be ongoing in some populations, and one aspect in favor of this is the genetic analysis of *C. albicans* strains isolated from members of mostly isolated communities. If *C. albicans* is indeed transmitted only vertically within humans, isolates from these remote communities with little outside contact should cluster tightly in a phylogenetic tree, which is not the case [10]. We hypothesize that the colonization rate increased upon introduction of a western life style (as suggested by the evolution experiment), which may have enabled more frequent vertical transmission by these adapted strains. At the same time, given the now-established presence of *C. albicans* in the environment [6], which we show here may already be adapted to humans, we propose that it is possible that humans sometimes acquire new strains from such environmental niches.
In short, based on our and others' findings, we do speculate a bit in this manuscript on alternative origins of colonizing *C. albicans* strains, but maybe not to the extent that the reviewer assumes here; we now have indicated this as a potential possibility in the discussion section to bring these points across more clearly (**line 580**)

There is some lack of context in the comparisons between SC5314 and the oak isolates. Figure 2 uses several other human isolates, and this is useful, as it shows that SC5314 is the outlier in some respects and Oak 1 and Oak 3 are outliers in others. But most experiments don't include other human isolates. Are the oak isolates unusually nutrient restricted or do they have more divergent ECE1 sequences? This would be useful information to put these strains on a more global phenotypic map.

Response:
We thank of Reviewer #1 for this constructive criticism. We have now included all clinical isolates in the growth assays (Figure 1) and added data to Supplementary Figure 1 to further show where the oak tree isolates differ from the other strains in the revised manuscript.
To address the reviewer's comment regarding *ECE1* sequences, we aligned the candidalysin amino acid sequence of the oak tree isolates with the known candidalysin sequences from 182 *C. albicans* isolates [11]. Interestingly the sequences of Oak 1 and Oak 2 are clearly distinct from all described sequences further supporting their different origin. We added this alignment in Supplementary Figure 2E (and below). Moreover, we added a

paragraph in the text (**line 190-194**) highlighting that the oak tree isolates have more divergent Ece1 sequences: "In Oak 1 and Oak 2, we identified new amino acid sequence variants of CaL (Suppl. Figure 2E), when comparing it with the known variants from 182 clinical *C. albicans* isolates [11]. The amino acid sequence of Oak 3 is analogous to one of the most common candidalysin variants identified in clinical isolates (Suppl. Figure 2E) [11]."
Furthermore, we elaborate on these findings in the discussion in **line 451-454** of the revised manuscript.

E

C. albicans candidalysin (CaL)	Amino acid sequence
SC5314 CaL – Variant A	SIIGIIMGILGNIPQVIQIIMSIVKAFKGNK
C. alb CaL – Variant B	SIISIIMGILGNIPQVIQIIMSIVKAFKGNK
C. alb CaL – Variant C	SIIGIIMGILTNIPQVIQIIMSIVKAFKGNK
C. alb CaL – Variant D	SIIGIIMGILGNIPQVIQIIMSIVRAFKGNK
C. alb CaL – Variant E	SIISIIMGLLGNIPQVIQIIMSIVKAFKGNK
C. alb CaL – Variant F	SIISIIMGILTNIPQVIQIIMSIVRAFKGNK
C. alb CaL – Variant G	SIISIIMGILSNIPQVIQIIMSIVRAFKGNK
C. alb CaL – Variant H	SIIGIIMGLLTNIPQVIQIIMSIVRAFKGNK
C. alb CaL – Variant I	SIIGIIMGLLTNIPQVINIIMSIIKAFKGNK
C. alb CaL – Variant J	SFLSIITALLGNIPQIIQIIMGIVKAFRGNK
C. alb CaL – Oak 1	SIISIIMGILTNIPQVIQIIMGIVRAFRGNK
C. alb CaL – Oak 2	SIISIIMGLLGNIPQVIQIIMSIVKAFRGNK
C. alb CaL – Oak 3	SIISIIMGILTNIPQVIQIIMSIVRAFKGNK

New *Supplementary Figure 2E:* Candidalysin sequences from oak tree isolates compared to clinical isolate sequences (taken from [11]). Different amino acids are indicated in purple. Oak 1 and Oak 2 have unique candidalysin sequences, Oak 3 has the same sequence as variant F, found in clinical strains.

In Figure 1A-B, the growth rates relative to SC5314 are presented as a heat map. The legend says the color coding is based on the p-value, but the scale is more consistent with it being based on a ratio of growth rates, and this matches the text as well. This should be clarified.

Response:
The original color code did, in fact, show the p-value. We have followed the suggestion of the reviewer and changed the color code to now show the growth relative to SC5314, with the significance indicated by asterisks.

When there is a significant difference in hyphal morphology, as there is within macrophages (Fig. 4), CFU plating is not a reliable means to quantitate cell number. One cannot conclude from this data that Oak 1 survives better in macrophages, it just forms individual yeast rather than multinucleated hyphae. With this sort of morphology difference, a metabolic assay (XTT), enumeration of nuclei, or qPCR for fungal DNA would be more accurate choices.

Response:
The reviewer is right, and we are aware of the CFU number being affected by the morphology. For initial survival, hypha formation does not play such a major role in macrophage interaction [12, 13]. Hence, this is an established method to quantify initial intracellular survival of *C. albicans* in macrophages at early time points, as shown here by others (*e.g.* [14-16]) and also in our studies (*e.g.* [17] Figure 2F, [18] Figure 4I) However, to further support our data, we have now confirmed our CFU data by staining and counting intracellular fungal cells 3 h post infection of monocyte-derived macrophages (Suppl. Figure 8D, E in the revised manuscript). By counting the fungal nuclei per macrophage (Suppl. Figure 8D and shown below), we removed the potential error of comparing yeast *vs.* hyphae with CFU plating, and confirmed our findings, showing that all oak tree isolates survived significantly better in macrophages compared to SC5314.

D

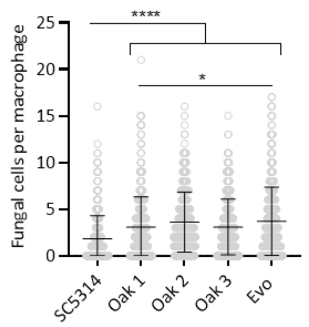

New Suppl. Figure 8D: Fungal cells per macrophage were determined 3 h post infection by staining fungal cells (ConA-AlexaFluor647, blue) and cell nuclei (DAPI, purple). Images were taken at 63× magnification with immersion oil (scale bar, 20 µm), and fungal cells per macrophage were counted. Each dot represents one macrophage. Infected macrophages from 4-6 donors were counted. Significance was calculated by using a one-way ANOVA with Tukey's multiple comparisons test (* p<0.05, **** p<0.0001).

In the same experiments, assaying macrophage survival at 24 hours is problematic given the literature that shows that these cells die from glucose depletion well before that (this is also apparent in Fig. S7, where the background macrophage damage rate is ~60%). This should really be done at shorter (~6h) timepoints.

Response:
It is true that macrophages die after *C. albicans* infection due to glucose depletion. However, the main pathways of *C. albicans* to induce macrophage lysis are *via* proinflammatory cell death induction (which is partially also

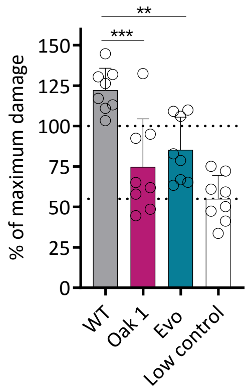

driven by the glucose depletion) [15, 19, 20] (ref), *via* the physical force of the growing hypha [21] and *via* candidalysin-induced damage ([17, 22], reviewed in [12, 13, 20]). For all these mechanisms, it is standard procedure and well-accepted in the literature to measure LDH release of unprimed macrophages 24 h post infection [17]. For LPS-primed macrophages, earlier time points are definitely necessary (*e.g.* [17, 23, 24]). The high damage level of the uninfected control (background damage) is not due to glucose depletion from the fungal infection, but is rather due to the fact that the cells are primary cells isolated from humans. Some donors can have a lower level of dying macrophages when they are uninfected (of approx. 30%), and some reach higher levels of about 80%. This is also shown in the figure below in the low control (taken from Suppl. Figure 8):

Suppl. Figure 8A: LDH release measured after 24 h. Each dot indicates one donor. The low control is the uninfected control.

In addition, to better visualize the donor variance, we have now modified all graphs in the revised manuscript of the macrophage LDH assays and intracellular survival data so that each dot represents one donor. We also re-named the "background damage control" to "uninfected control" in all figures, as this term otherwise implies that some damage is induced to those samples, but it rather shows the natural turn-over of the cells during the experiment.
To show the damage kinetics of the oak tree strains and the evolved strain better, we performed live-cell imaging with monocyte-derived macrophages and propidium iodide staining. These new data (shown below) fully support our finding that the oak tree strains induce less damage than SC5314, and are now included in the revised manuscript as the new panel B to Suppl. Figure 8. The data further demonstrate that the infection kinetics are slower than the reviewer anticipated: several oak-tree isolates had not initiated measurable host-cell damage by six hours post-infection, precluding detection of strain-specific differences at that early time point. Additionally, the minimal cytotoxicity observed in the uninfected controls across all four donors confirms that macrophage death is not attributable to glucose depletion. These findings justify extending the assay to a 24-hour endpoint, by which time the reference strain SC5314 has reached its maximal macrophage-killing capacity, whereas the environmental isolates continue to exhibit progressive cytotoxicity.

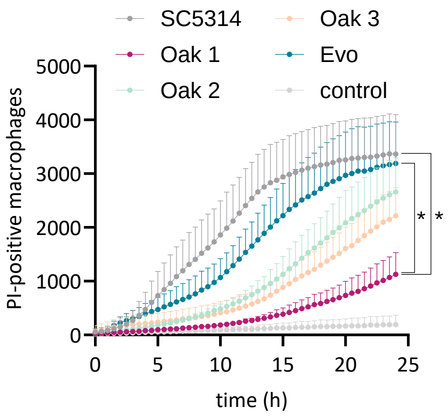

New *Suppl. Figure 8B:* Host cell lysis of monocyte-derived macrophages was measured by propidium iodide staining over the course of 24 h. Significance was calculated using a two-way ANOVA with repeated measures and Tukey's multiple comparison test (* p<0.05) (4 donors).

The "evolution" experiment identifies no genomic changes that are worth elaborating upon, but a considerable transcriptomic change. How do the authors imagine this happening? Epigenetic? Some discussion is warranted.

Response:
Our genetic analysis used a very conservative approach to reliably detect mutations in the diploid genome, while avoiding false positives. While we did not find a "smoking gun" in the form of a missense or nonsense mutation in the ORF of a potentially relevant gene, we detected some intergenic and silent mutations that may affect the phenotype *via* more subtle regulatory activity. We added an elaborative sentence in the main text in **line 284** of the revised manuscript.
We can also not exclude epigenetic effects, or the possibility that we accidentally filtered out a relevant mutation in our pipeline. We have now added an additional section to the discussion to discuss some of these possibilities (**line 507-511**). In any case, as the data were not as clear-cut as we had hoped, we did not follow it up with further in-depth analyses, which would not have contributed much to the overall findings of this paper.

The statistical analysis is incomplete in places. Data for the Oak strains are compared to SC5314, but in the experiments with the Evo strain it should be compared to the parent as well.

Response:
This is a fair point. In fact, we have always performed the statistical comparison between the Evo strain and its parent. However, the differences were frequently not significant and, therefore, not indicated. To clarify this in the revised manuscript, we have added a sentence in the methods section under "Statistics and Reproducibility" (**line 844**).

Fig. S8 is important. There are really no differences in metrics of immune activation amongst these strains. This argues that the differences in outcomes (invasion, hyphal growth, damage, etc.) are fungal-intrinsic, i.e., due to strain variation rather than differential recognition by the macrophages. This should be emphasized more in the main text in the discussion around Fig. 4.

Response:
We fully agree. As suggested, we now emphasize this more in the results section and in the discussion of the revised manuscript. Specifically, we have added a paragraph in the results section (**line 236-240**): "To investigate whether these different phenotypes are driven by the macrophages or due to the fungal-intrinsic strain variations, we investigated the immune activation of the oak tree isolates with macrophages (Suppl. Figure 9B). Interestingly, all three strains did not differ significantly in their activation of IL-8, TNFα, IL-6, and IL-1β release, except for Oak 3, which spiked a significantly higher level of IL-8."
Furthermore, we added a corresponding statement in the discussion (**line 429**): "[…] This high virulent phenotype seems to be fungal-intrinsic, as the reaction of immune cells to all oak tree isolates remained the same and is likely attributed to strain variation rather than differential host responses. […]"

Reviewer #2:
Lange et al. have analyzed three environmental isolates of Candida albicans originally isolated from oak trees in the United Kingdom. They determined the differences between these isolates and the standard C. albicans isolate SC5314 for many properties including host damage, hyphal formation, cellular invasion, and production of candidalysin, a C. albicans protein that causes cell damage. One strain behaved similarly to clinical isolates while the other two were less virulent. The more virulent strain had reduced susceptibility to the antifungal amphotericin B, while all three strains had a reduced susceptibility to fluconazole. One less-virulent strain was evolved over months on galactose, a sugar that is part of the human diet. The evolved strain became more virulent, more metabolically flexible, more able to damage epithelial cells, and more resistant to Amphotericin B. This suggests that virulence and antifungal resistance can be evolved in an environmental strain, and that adaptation to sugar rich diets can affect virulence and drug resistance.

The manuscript is well written. The data for the most part of well-presented and the interpretations are valid without overinterpretation. The conclusions are justified.

Response:
We thank this reviewer for their time and for this positive and encouraging feedback!

Reviewer #3:
The authors characterized environmental Candida albicans isolates and show that evolution in presence of dietary sugars of a non-virulent strain results in a more virulent strain. They also show that such isolates may already be resistant to antifungal drugs.

The authors used various approaches to make their point that non-virulent strains may become virulent under environmental pressure conditions.
It is mainly a descriptive manuscript were the molecular basis of the different phenotypes are not really explored.

The paragraph on the possible role of Ece1 and its expression level is hypothetical and the possible mechanisms (TATA box location or presence) have not been explored. Also the secretion of the peptides does not really explain the higher damage potential of oak2. This whole part is rather speculative.

Response:
In reply to the role of secreted peptide, we would like to stress that the peptide in question is the well-established *C. albicans* peptide toxin, candidalysin. Since our own published data show that candidalysin is the key damaging factor of *C. albicans* [25], we hypothesized that the high damage potential of Oak 2 could be associated with strong expression of *ECE1* and candidalysin peptide production. This was in fact what our data showed: *ECE1* was expressed at high levels in Oak 2 on epithelial cells, which we could show by the detection of this peptide toxin in supernatants of Oak 2 (Figure 3D). We also showed that Oak 2 has the potential to invade into epithelial cells at a similar rate as the strongly damaging control strain SC5314 (Figure 2E). As recently shown by our laboratory [26] a combination of these phenotypes – high levels of *ECE1* expression, candidalysin secretion, and invasion into epithelial cells – allows candidalysin delivery to human cell membrane and thereby host cell damage. These phenotypes therefore readily explain the high damage potential of Oak 2. This has now been more carefully explained in the revised manuscript (**line 445-451** and **line 467-471**).
Concerning the TATA box, we agree that this section is hypothetical, which is why we phrased the possible relevance carefully ("potentially explaining the different expression dynamics") and provided the data mainly in form of supplementary figure. To address this comment, we have changed the text to a more careful phrasing (**line 462**). This attempt at an explanation is part of a section with information on the Ece1 sequence and its promotor region that we believe is of interest to many readers, given the link between host cell damage and candidalysin expression. A deeper exploration of the potential relevance of the TATA box was therefore not planned, especially as the regulation of *ECE1* is very complex and difficult to disentangle in a non-model strain of *C. albicans*. The mechanistic explanation for the damage therefore is the functional invasion and the high candidalysin expression, but we agree that further research remains to be done in the future.

Part II - Major Issues: Key Experiments Required for Acceptance

Reviewer #2:

1. Evolving a strain over months requires precise sterile techniques. Is it clear that the Evo strain and the Oak2 strain are the same strain with minor changes. Can you rule out a different strain contaminating the culture. It is important to state that.

Response:
We are confident that we can exclude contaminations with a different *C. albicans* strain during the evolution experiment. Firstly, we have used protocols based on our previous evolution experiments (*e.g.* [27]) and performed all experimental procedures in this evolution experiment carefully under sterile conditions.
Secondly, we sequenced both the initial, parent strain (Oak 1, we assume this one is meant by Reviewer #2) and the evolved strain after concluding the evolution experiment, where our (admittedly very conservative) screen for mutations found that the genome sequences are very similar with few changes. As Oak 2 is a strain representing a new, previously unknown clade [5], a contamination with a common lab strain is very unlikely, as this would have shown a significant number of genetic differences. The combination of genetic near-identity with subtle phenotypic differences lets us be reasonably certain that we are investigating a strain before and after adaptation.

2. Fig 5A is very confusing. The left and center flasks are clear. What is the third flask and what does it represent? What is meant by adaptation and selection for the right flask? Isn't that going on in the center flask? The figure says samples are taken from the center and right flasks every 3 days. How is the right flask different from the center flask. This needs to be explained better in the text and in the figure legend.

Response:
We agree with this comment and have changed Fig. 5A and the legend accordingly, as shown below.

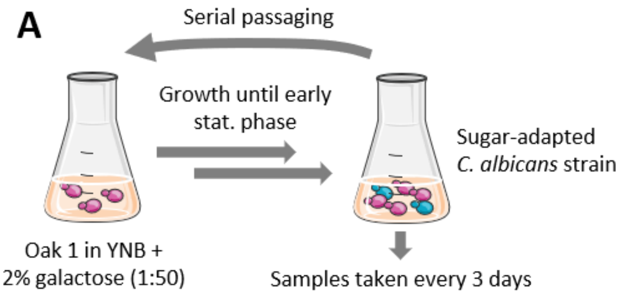

New Figure 5A: Schematic overview of the evolution experiment in YNB medium supplemented with 2% galactose. Oak 1 and SC5314 overnight cultures were diluted 1:50 into the galactose-containing medium and were incubated for 6 weeks at 30 °C, with passaging them in fresh medium every 2-3 days after the early stationary phase was reached. Growth was measured as OD_{600nm} over the whole time course. Samples were taken and frozen after every passage.

3. The text needs to more closely reflect the growth conditions in the Figure 1,

Response:
We agree that we may have glossed over some important details in this section and thank the reviewer for pointing that out. We now describe the observed growth with different nutrients in more detail in the results section in **lines 113-124**.

4. As this is PLOS Pathogens, it would seem necessary to show the behavior for the strains in a OPC or systemic animal model. Maybe competition in either model rather than showing each strain separately. At least OAK2 and EVO separately or in competition.

Response:
We somewhat disagree with the notion that any manuscript in *PLOS Pathogens* must necessarily include animal models, and would like to point out that, for example, many interesting and important publications without such models (for example [28-35]) can be found in the latest issues of *PLOS Pathogens*. We have here decided to use *in vitro* infection models for good reasons. Using *in vitro* and *ex vivo* systems to mimic fungal infections of various human (*versus* mouse) cell types and their advantages have been extensively discussed in recent reviews [36, 37]. In the present study we have used simple epithelial monolayer models, which clearly showed the different virulence potentials of the strains. We also used primary human macrophages as a simple model for the initial immune interactions. We then expanded on these first finding using more complex models that importantly are all based on human, and not murine cells, and which are nearer to the physiological conditions during infections then the simpler models: a gut-on-chip model established in our laboratory [38, 39] and an *ex vivo* human whole blood infection model [35, 40]. Following the 3Rs principle (Replace, Reduce, Refine) as a framework for ethical animal research, we would argue that additional animal experiments would not be not required to support our main conclusions.
We also noticed that the competition experiment was suggested between Oak 2 and the (Oak 1-evolved) Evo strain. As these two strains have no relation to each other, we assume that there has been some misunderstanding on the relationships of the strains. To avoid confusions for readers and to present the data more consistently, we have therefore decided to remove Oak 2 from the gut-on-chip data shown in Figure 8, and have placed it into a separate supplementary figure (new Suppl. Figure 7).

Reviewer #3:
The oak 2 strain shows many characteristics of a virulent strain. How can it be excluded that this is a 'contaminant' strain. Can such strains be reisolated from that oak tree? A phylogenetic tree that places the three oak strains among sequenced isolates from the different Candida clades should be done.

Response:
We can exclude that Oak 2 is a contamination. Our manuscript is based on the 2019 work of Bensasson *et al*. in Genetics [5], who isolated the Oak tree strains used in our study. They extensively discussed the original habitat of these isolates and compared the corresponding genome sequences with clinical isolates. To cite from this work: "Here, we generated genome sequences for three strains of *C. albicans* from oak bark from the New Forest in the United Kingdom (Robinson *et al.* 2016). These are the first *C. albicans* genomes sequenced from a nonanimal source, and we compared them to the genomes of over 200 strains from humans and animals (Muzzey *et al.* 2013; Hirakawa *et al.* 2015; Ropars *et al.* 2018). Although they were collected from the same woodland, these oak strain genomes are genetically diverged from one another, and are more similar to diverged clinical lineages than they are to each other. However, oak strains differ from clinical strains in showing higher levels of genome-wide heterozygosity, **suggesting that they were subject to different selection or mutation pressures.** The genetic diversity of *C. albicans* in this woodland implies that they have **lived there long enough for distinct lineages to accumulate** on unusually old trees. Therefore, *C. albicans* can live **in the oak environment for prolonged periods**."
Based on these previous investigations, we are rather confident that the strains can be considered "environmental", as in contrast to "human-associated". We have changed the text to better describe the environmental nature of the isolates and their origin by more extensively referring to the original publication in the revised manuscript (**line 421**, **line 487-496**).
Bensasson *et al*. [5] analyzed the corresponding genome sequences in comparison with over 200 strains from humans and animals and the phylogenetic tree showing the position of the oak trees in comparison to those clinical isolates can be found as Figure 2A in Bensasson *et al*. [5].

The authors write that growth on glucose is very different between WT and the oak strains, but on YPD (supplementary figure 1), this is not clear. What may be the reason.

Response:
We suggest that this is due to the ability of *C. albicans* to effectively use a very broad range of nutrients. Since YPD is a rich medium that contains a multitude of compounds in addition to glucose, including multiple peptide sources, there may be multiple reasons for growth effects. These additional components are not included in the defined minimal medium that was used for the growth assays with glucose (Yeast nitrogen base + glucose + ammonium sulfate), and altogether likely masked the less efficient use of glucose by the oak strain.

It is also unclear why for AMB and FLU different conditions are used (30 ° for 2 days versus 37 °C for 6 days). I think that the FLU data should also be in figure 1 with data shown at the same temperature and same time points.

Response:
We thank the reviewer for these suggestions, and we have now moved the fluconazole data to Figure 1 in the revised manuscript.
Regarding the different temperatures and time points: We tried different temperatures that not only reflect body temperature but also the environment. We found interesting phenotypes for these tested antimycotics at these specific temperatures and time points (see figure below).
To address the reviewer comment, we added the drop tests of fluconazole at 37 °C after 6 days and with amphotericin B at 30 °C after 2 days to the manuscript (added to Figure 1). Notably, we did not observe any growth for all strains in presence of AmB when using the same incubation time and temperature as with fluconazole.

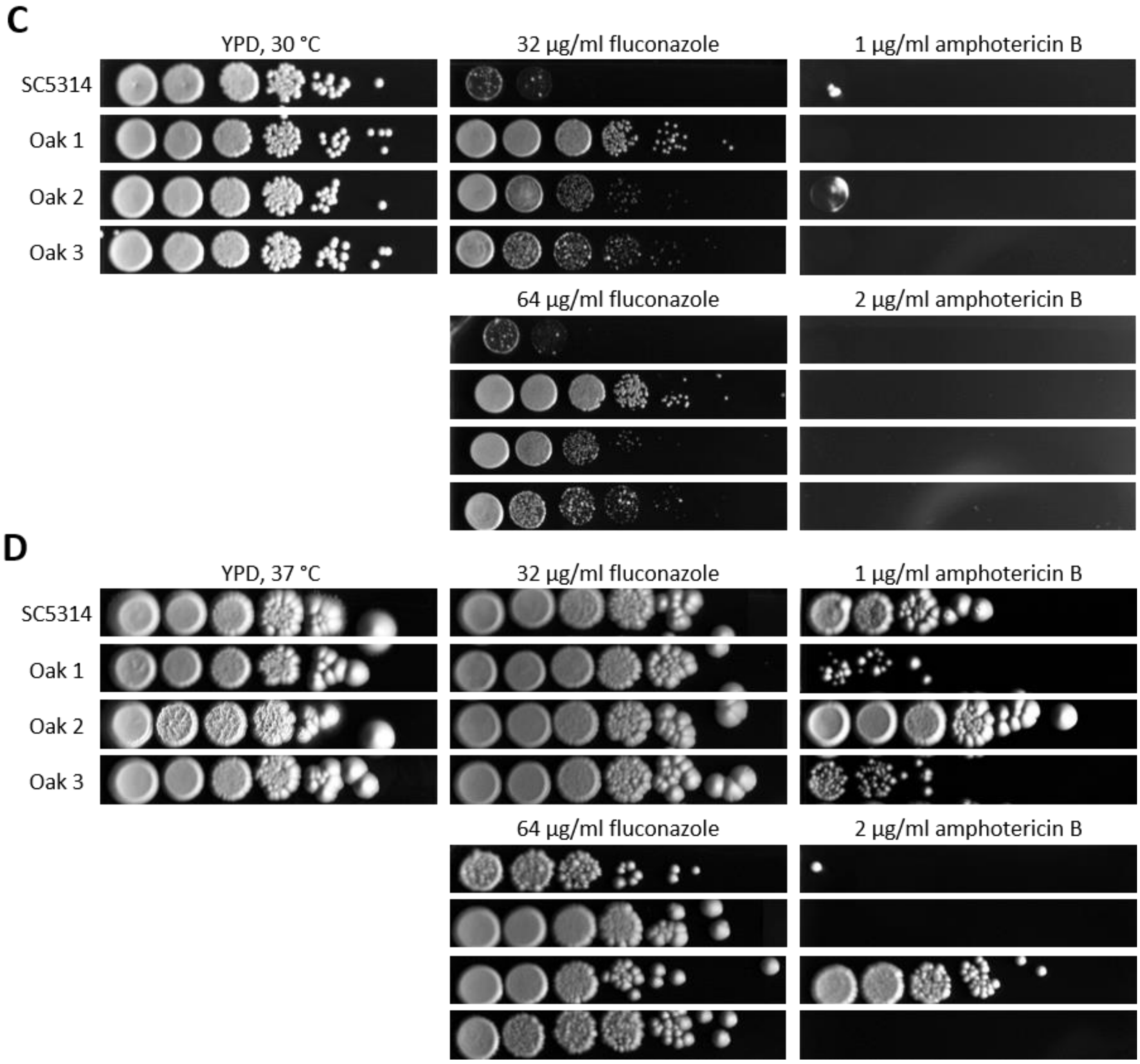

New *Figure 1C, D*: Comparison of different drop test incubation times and temperatures.

More importantly here, Figure 1 has been performed at 30 °C but data should be provided at 37 ° C in order to translate better to the human condition.

Response:
There are two reasons why we have opted to perform the growth tests at 30°C. First, we are testing environmental isolates here, which we assumed to be adapted to lower temperatures than the human body. For a direct comparison among them, we therefore used 30°C as a compromise between the unknown environmental temperature (likely lower) and the 37°C of the human body. This also allowed sufficiently fast growth, which is part of the second, more technical reason. From experience, we know that *C. albicans* readily forms hyphae at 37°C (especially in media limited for some nutrients), and these hyphae would disrupt our growth measurements by their uneven distribution and their linear, rather than exponential growth pattern. At 37°C, any difference in hyphae formation (for example seen with Oak 1) would quickly occlude the growth speed differences we aimed to determine. As such, 30°C is maybe an imperfect growth condition for aspects of the human environment, but the best and most frequently used temperature in these kind of comparative assays on metabolic capacities.

What is the molecular mechanism of the higher AMB resistance of the Oak 2 strain? This should be the result of a mutation. What does the whole genome sequnce of this strain says?

Response:
We thank the reviewer for these suggestions, and we agree that it would be of interest to know the molecular mechanism, but this complex question is likely not easy to answer. The Oak strains deviate from the SC5314 reference genome substantially, so an untargeted search for mutations is not a feasible approach within the scope of this manuscript. However, we have extracted the sequences of *UPC2*, *MDR1*, *ERG2*, *ERG3*, *ERG5*, and *ERG11* from the sequence data deposited by Bensasson *et al*. [5] to look for typical mutations associated with resistance. We could identify a frameshift mutation in both alleles of the *UPC2* gene in the Oak 2 strain, which likely lead to a loss-of-function of the encoded protein (as they scramble much of the activation domain). Upc2 regulates ergosterol biosynthesis genes and deletion of *UPC2* leads to reduced levels of ergosterol [41, 42], the target of amphotericin B. Thus, this could potentially influence the amphotericin B susceptibility of Oak 2.
We added a paragraph in the discussion about these two mutations in *UPC2* (**lines 474-479**) with a reference to the data set by Bensasson *et al*. [5].

The oak strains grow less well in glucose medium. For the damage experiment on the epithelial cells, should more oak mutant cells be used compared to the WT. Over a 24 h period, this may result in a growth difference.

Response:
We agree that growth differences might contribute to a different fungal burden on the epithelial cells over the course of the infection, which in turn would affect damage induction. However, with our damage assays we aim to assess the complete virulence potential of the strains, which also includes the ability to grow on the host cells. Therefore, we would argue that this is not a problem, as we measure the ability of a specific inoculum of fungi to induce damage, all steps from growth to dedicated host cell destruction included.

However, we have now performed additional growth curve experiments with cell culture media (see figure below). We saw that there was no correlation between fungal growth in the media and their damaging potential during infections. For instance, DMEM F12 was used as medium for oral cell infections, and Oak 1 showed the best growth there, but it had significantly lower damage induction compared to SC5314 (see main text, Figure 2A).

Based on these data, we believe that the main factors that are important for damage induction to epithelial cells are filamentation, invasion, and candidalysin production, all of which we characterized in the oak tree strains.

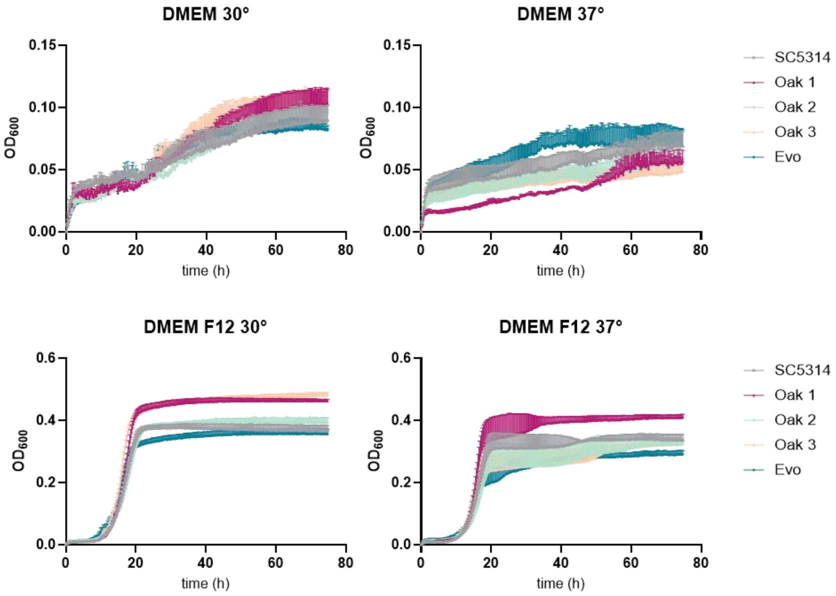

Figure for this response: Growth in cell culture medium – DMEM (intestinal cell infections) and DMEM F12 (oral cell infection). Growth assays were performed at 30°C (left) and 37°C (right).

For the evolution experiments in the text says YNB+Gal but in legend is SD+Gal.

Response:
We thank the reviewer for spotting this mistake; we have changed the text accordingly.

The authors evolved in galactose to mimick the sugar that may not be taken up in small intestine. However, there is also Candida in the small intestine.

Response:
We agree with the reviewer's assumptions. While the main reservoir of commensal *C. albicans* cells is in the large intestine, it is also present in the small intestine. The aim of our evolution experiment was to use a dietary sugar that is not taken up by the host and, thus, can be metabolized by *C. albicans*. As the sugar is not taken up in the small intestine, the *C. albicans* in both the small and large intestine should be able to metabolize this carbon source and evolve accordingly.

It would be interesting to also evolve the strain in glucose containing medium.

Response:
We agree that this would be an interesting experiment, which would, however, require a substantial amount of time and effort that seems not proportional to the additional insight for the specific questions of our manuscript. Our evolution experiments normally take between 2 and 3 months, and at least additional 2-3 more months to characterize the evolved strains. Although this and other variants of experimental evolution are interesting approaches to further characterize *C. albicans* adaptations to life in the human gut in the future, we believe that currently, this would be well beyond the scope of our manuscript.

Apparentely some evolved strains (being able to grow well in galactose) were not used further on, but only one 'evo' strain was used. What does the whole genome sequence tells regarding the differences with the parental strain?

Response:
We performed the sugar evolution in biological triplicates. As shown in Supplementary Figure 3, the strongest adaptation to the host environment was observed in Evo 3 (later called Evo), so we went on to characterize this strain. The whole genome sequencing revealed very little mutations for all three evolved strains (all showed in Suppl. Table 3). As we could not find a strong phenotype and no particular genomic change for the other two sugar-evolved strains, we excluded them from follow-up experiments.

The authors did this, but did not really find any interesting mutation and only one change in a coding sequence. This raises the question whether the evolved strains were passaged multiple times under control conditions to rule out that it were just adapted strains but not mutated strains?

Response:
We did not perform a control condition evolution experiment, however, so far the phenotype was always stable when re-thawing the strain and culturing it continuously on YPD plates and in culture. So we believe that this strain is in fact stable and, thus, likely mutated.

It is strange that a strain that did not grow on glucose nor galactose, was evolved in galactose and then starts to grow very well on glucose and still very poor on galactose. What is the molecular mechanism?

Response:
We assume that one or some of the detected genetic or possibly epigenetic changes in the strain led to the upregulation of sugar transporters such as *HGT17*, *HGT2*, *HGT7*, *HGT19*, *HGT1*, *MAL31* and others, and an upregulation of sugar sensors like *HGT4*. This is shown in the figure below, which is also depicted in Supplementary Figure 4C and described in the main text (results: **line 304-313**, discussion: **line 505-511**):

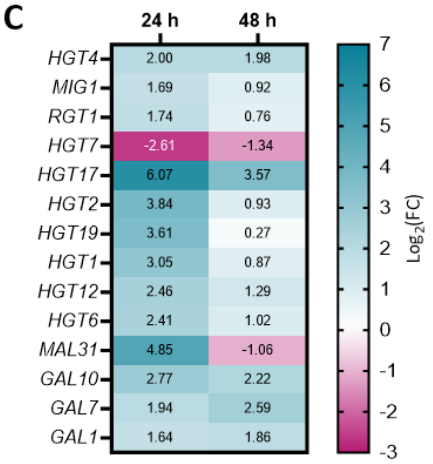

Gene	Function
HGT4	Glucose and galactose sensor
MIG1	Repressor; regulates genes for carbon source utilization
RGT1	Repressor; involved in the regulation of glucose transporter genes
HGT7	Putative MFS glucose and galactose transporter; expressed at high glucose levels
HGT17	Putative MFS family glucose transporter; induced at low glucose levels in rich media
HGT2	Putative MFS glucose transporter; expressed in rich medium with 2% glucose
HGT19	Putative MFS glucose/myo-inositol transporter; expressed in rich medium
HGT1	High-affinity MFS glucose transporter
HGT12	Glucose, fructose, mannose transporter
HGT6	Putative high-affinity MFS glucose transporter
MAL31	Putative high-affinity maltose transporter
GAL10	UDP-glucose 4-epimerase; galactose utilization
GAL7	Putative galactose-1-phoshphate uridyl transferase
GAL1	Galactokinase

Suppl. Figure 4C: Log_2(fold change) of genes involved in glucose and galactose sensing, transport, as well as in the downstream signaling pathways [43] of the sugar-adapted strain relative to its parental strain (oak tree isolate 1) on intestinal epithelial cells. Gene functions were taken from candidagenome.org.

We would like to stress, however, that we found increased growth several sugars, including galactose, where the latter only failed to reach statistical significance:

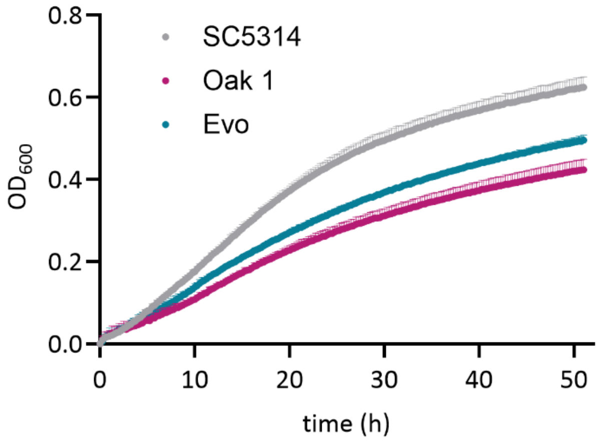

Figure for this response: Growth in YNB + 2% galactose. Growth curve experiments were performed at 30 °C (n = 3).

This is mentioned in the text in **line 254** (and also later in **line 502-505**) as rationale for the follow-up experiments: "…one of the evolved replicates, from here on indicated as 'Evo', adapted well to growth on galactose and was therefore chosen for further investigation."
Importantly, glucose is the preferred carbon source of *C. albicans* and metabolized much easier than galactose. That means that if import is enhanced (see Supplementary Figure 4), increased glucose uptake would most likely have a larger effect on growth than a similar increase in galactose uptake. However, under galactose-only conditions, even this smaller benefit would be selected for as a beneficial adaptation.

Part III – Minor Issues: Editorial and Data Presentation Modifications

Reviewer #1: (No Response)

Reviewer #2:

1. Fig 2F – why is Oak 3 not included in the analysis as it is included in all other panels4.

Response:

Based on this suggestion, we have now repeated the *ex vivo* whole blood experiment with the Oak 3 strain and included the data in Figure 2F of the revised manuscript as shown below:

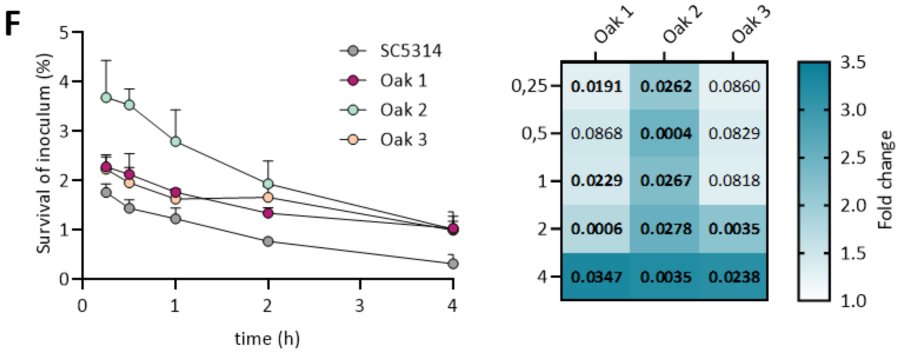

New *Figure 2F*: Survival of *C. albicans* in whole human blood was assessed over the course of 4 h by plating surviving colony-forming units (4 donors). Survival is shown relative to the inoculum. Heat map displays fold change to SC5314 and p-values calculated with a two-way ANOVA with repeated measures and Dunnett's multiple comparison test. Significant p-values are indicated in bold.

2. Line 95 – says that there was reduced growth in all texted alternative nitrogen sources? What about arginine where there is no reduced growth?

Response:

We thank the reviewer for spotting this small mistake and have changed the text accordingly to explicitly exclude arginine: "Moreover, Oak 1 showed deficits in growth on all tested alternative nitrogen sources, except for arginine (Figure 1A, B)." (**line 120-121**).

3. Line 97 – says that Oak 2 grew similarly to SC5314 on the texted nitrogen sources – what about Tryptophan and some concentrations of ammonium sulfate.

Response:

Similar to above, we have now changed the text accordingly: "Finally, Oak 2 grew similarly to SC5314 on the tested nitrogen sources (Figure 1B), with the exception of phenylalanine, tryptophan, and very low concentrations of ammonium sulfate." (**line 122-124**).

4. Line 201 – There is no Figure 5E.

Response:

We thank the reviewer for pointing that out and changed the text accordingly. We removed the reference to Figure 5E, which does not exist, in **line 263 and 267**.

Reviewer #3:

Why is the interaction with the intestinal epithelial cells for 48 h and the oral ones for 24hrs.?

Response:

This is common practice in these infection models: Intestinal cells (in our case a mix of intestinal epithelial and goblet cells) are less sensitive to *C. albicans* damage induction due to the structure and the mucus layer, which prevents induced endocytosis and only allows active penetration [44]. On oral cells, *C. albicans* invades *via* both, active penetration and induced endocytosis [44], which leads to a higher percentage of invading hyphae, and candidalysin-induced damage. Moreover, intestinal epithelial cells are more resistant to candidalysin-induced

damage [45]. This normally necessitates a longer infection before damage becomes visible, reflecting the natural differences between host cell types.
The low release of LDH on intestinal cells even with a high damaging strain like SC5314 makes it very difficult to see difference in damage induction among strains with a low virulence. These often fall below the detection limit/background damage. Thus, we increased the incubation time for the LDH assays on intestinal cells.
To address this comment, we added a small explanation in the main text (**line 147**): "Host cell damage was determined *via* lactate dehydrogenase (LDH) release 24 h and 48 h after infection of oral or intestinal epithelial cells, respectively, to account for the different infection kinetics with the different host cells (Figure 2A, B)."

In figure 2F, is it correct that survival in whole blood is only 2-4 % after 15 min? Why is strain oak3 not presented here?

Response:
As this was also suggested by Reviewer #2, we performed the whole blood experiment also for Oak 3 and have now included this new data in Figure 2F. We found the low survival rate consistently also with Oak 3.

Strain Oak3 is also missing in figure 3D.

Response:
The rather complex and time-consuming HPLC-MS measurements were performed only for the strains at the focus of this publication, Oak 1 and Oak 2. While we agree that it would give a more complete picture for Oak 3, we do not think this data necessary for the overall conclusions in this manuscript. Therefore, we have opted to not perform the additional measurements, but we have included a sentence in the figure legend to point out this fact (**line 1170**).

Panel C is not indicated in the legend of figure 4.

Response:
We thank the reviewer for pointing that out and have changed the figure legend accordingly (**line 1179**).

In 4C, I do not see the monocytes in the panel of SC5314. Are they already destroyed by the Candida? Oak1 and Oak3 are not able to escape?

Response:
The macrophages are still present in the SC5314 sample, however, they are not readily visible in this focal plane. For a visual guide, we have added outlines in the figure below for some of the macrophages. We do agree that it remains difficult to see and have therefore also included fluorescent microscopy pictures to Figure 4 and Supplementary Figure 8 in the revised paper, where the macrophages are readily visible.

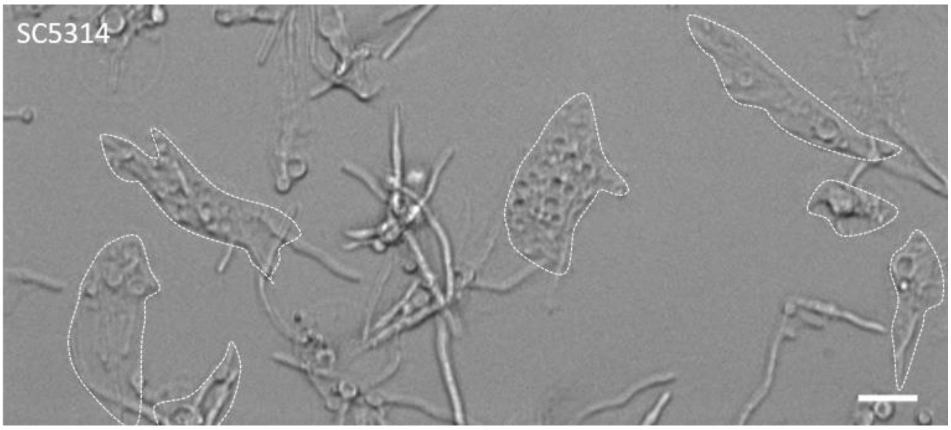

Figure for this response: Microscopy images from former Figure 4C (now moved to Suppl. Figure 8). Scale bar: 20 µm. Dashed outlines indicate some of the macrophages.

Oak 1 and Oak 3 are indeed capable of escaping the macrophages. However, as these two strains are less capable to filament inside macrophages, their escape is delayed in comparison to SC5314. In contrast to the hypha-driven main mechanism of SC5314 and Oak 2, this likely happens by macrophage bursting after Oak 1 or Oak 3 have reached a high enough fungal load *via* intracellular replication.

Would it not have been interesting to take the most virulent Oak strain in the evolution study. The effect might even be bigger

Response:
We agree that this would be also be an interesting approach. Our rationale for the experiment presented in the manuscript was however that an evolution experiment may increase the virulence of a non-adapted strain with low virulence, Oak 1. To clarify this, we added a small sentence in the main text (**line 244**): "Oak 1 has shown diminished growth with all tested host dietary sugars and a low virulence potential, which feasibly could be increased if the strain were to adapt better to the human host."
As described in an answer above, many more evolution experiments would be of interest to characterize the adaptation mechanism, but limited resources required us to focus on the most promising. In the future, this may be an interesting complementary approach.

Is the MIC for Flu or Amb changed in the evolved strain?

Response:
Yes, they are indeed changed in the evolved strain, as it exhibits a higher MIC50 in both, fluconazole and amphotericin B, compared to its parent Oak 1. The MIC50 numbers, according to the EUCAST protocols, are depicted for the strains in the table below.

Strain	AmB MIC50 (µg/mL)	Fluc MIC50 (µg/mL)
SC5314	0,125	0,0625
Evo	0,5	>4
Oak 1	0,125	0,125
Oak 2	0,25	0,5
Oak 3	0,25	0,125

In line 275 it is indicated that ECE1 expression is not changed, but then 10 lines further major conclusion is ECE1 expression is increased in evolved strain after 3 hours. Was it known that the transcript of ECE1 is not stable?

Response:
The first statement in line 275 (now **line 339**) was about *ECE1* expression at later stages of infection, *i.e.* 24 h and 48 h post infection. The main peak of *ECE1* expression *in vitro*, however, is earlier – roughly after 3 h [46]. A high expression of *ECE1* at this 3 h-time point is known to be necessary for damage induction to host cells [26].
We added now something to this results section (**line 339**): "All other hypha-associated genes, however, such as *ALS3*, *ECE1*, or *HWP1* did not differ in their expression. This suggested that the increased damage by the sugar-adapted strain at these later infection stages is mainly driven by its enhanced filamentation and not by an increased transcription of hypha-associated genes.
And in **line 345**: "We additionally examined the expression of *ECE1* at 3 h, when the *ECE1* transcript levels are the highest *in C. albicans*, which is known to be necessary for damage induction to the host [26]."

It is quite surprising to see the major differences between in vitro data and the on chip data (which are actually also in vitro).

Response:
The gut-on-chip is a more complex system, which includes immune system components, a 3D-structure, and flow [38, 39]. Nevertheless, it was quite surprising that the observed effects were not as strong as in our other *in vitro* experiments but showed a similar trend. We believe that in the more complex organ-on-chip system, the effects may have been masked by other interactions, which are difficult to disentangle. In our view, this shows the value of using models of different complexity levels to obtain a fuller picture.
To address this concern, we added an elaborative sentence in the main text (**line 377**): "…supports the previously observed increase in its virulence (Figure 8D) and suggests a galactose-driven adaptation to the gut niche, which among our models is best simulated by this organ-on-chip system."

How far away is the oak tree from the nearest field where agricultural azoles may have been used?

Response:
This is an interesting question. The latitude and longitude of the isolation spots on the oak trees were described by Bensasson *et al*. [5]. Interestingly, some trees were quite close to fields [5], which is also why we mentioned the probability of the oak tree isolates to acquiring their resistance through the presence of agricultural azoles. We now added a sentence to the discussion (**line 475**): "Exposure of environmental fungi to to agricultural azoles has been suggested as a cause for resistance development [47, 48], and this cannot be excluded here: The isolates were found at isolation sites that are not too far from agriculturally used areas [5]."

Literature cited in these responses

1. Emmons CW, Histoplasmosis: animal reservoirs and other sources in nature of pathogenic fungus, *Histoplasma.* American Journal of Public Health and the Nations Health, 1950. **40**(4): p. 436-440.
2. Brito MdO *et al.*, Isolation of Cryptococcus species from the external environments of hospital and academic areas*.* The Journal of Infection in Developing Countries, 2019. **13**(06): p. 545-553.
3. Nielsen K, De Obaldia Anna L, and Heitman J, *Cryptococcus neoformans* Mates on Pigeon Guano: Implications for the Realized Ecological Niche and Globalization*.* Eukaryotic Cell, 2007. **6**(6): p. 949-959.
4. Soltani M *et al.*, Isolation of *Cryptococcus neoformans* and other opportunistic fungi from pigeon droppings*.* Journal of Research in Medical Sciences, 2013. **18**(1): p. 56-60.
5. Bensasson D *et al.*, Diverse Lineages of *Candida albicans* Live on Old Oaks*.* Genetics, 2019. **211**(1): p. 277.
6. Opulente DA *et al.*, Pathogenic budding yeasts isolated outside of clinical settings*.* FEMS Yeast Research, 2019. **19**(3).
7. Robinson HA, Pinharanda A, and Bensasson D, Summer temperature can predict the distribution of wild yeast populations*.* Ecology and evolution, 2016. **6**(4): p. 1236-1250.
8. Amsri A *et al.*, Adaptation to an amoeba host drives selection of virulence-associated traits and genetic variation in saprotrophic *Candida albicans.* Frontiers in Cellular and Infection Microbiology, 2024. **14**.
9. Morio F, Dear medical mycologists, it is time to look outside the box*.* FEMS Yeast Research, 2020. **20**(1): p. foz080.
10. Angebault C *et al.*, *Candida albicans* Is Not Always the Preferential Yeast Colonizing Humans: A Study in Wayampi Amerindians*.* The Journal of Infectious Diseases, 2013. **208**(10): p. 1705-1716.
11. Wickramasinghe Don N *et al.*, Variations in candidalysin amino acid sequence influence toxicity and host responses*.* mBio, 2024. **15**(8): p. e03351-23.
12. Lange T *et al.*, "Under Pressure" – How fungi evade, exploit, and modulate cells of the innate immune system*.* Seminars in Immunology, 2023. **66**: p. 101738.
13. Sonnberger J *et al.*, "We've got to get out"—Strategies of human pathogenic fungi to escape from phagocytes*.* Molecular Microbiology, 2024. **121**(3): p. 341-358.
14. Nicola André M *et al.*, Macrophage Autophagy in Immunity to Cryptococcus neoformans and Candida albicans*.* Infection and Immunity, 2012. **80**(9): p. 3065-3076.
15. Uwamahoro N *et al.*, The Pathogen *Candida albicans* Hijacks Pyroptosis for Escape from Macrophages*.* mBio, 2014. **5**(2): p. e00003-14.
16. Salvatori O *et al.*, Bacteria Modify Candida albicans Hypha Formation, Microcolony Properties, and Survival within Macrophages*.* mSphere, 2020. **5**(4): p. 10.1128/msphere.00689-20.
17. Kasper L *et al.*, The fungal peptide toxin Candidalysin activates the NLRP3 inflammasome and causes cytolysis in mononuclear phagocytes*.* Nature Communications, 2018. **9**(1): p. 4260.
18. Ding X *et al.*, Inflammasome-mediated GSDMD activation facilitates escape of *Candida albicans* from macrophages*.* Nature Communications, 2021. **12**(1): p. 6699.
19. Wellington M, Koselny K, and Krysan DJ, *Candida albicans* morphogenesis is not required for macrophage interleukin 1beta production*.* mBio, 2012. **4**(1): p. e00433-12.

20. Krysan DJ, Sutterwala FS, and Wellington M, Catching Fire: *Candida albicans*, Macrophages, and Pyroptosis. PLOS Pathogens, 2014. **10**(6): p. e1004139.
21. Westman J *et al.*, Lysosome Fusion Maintains Phagosome Integrity during Fungal Infection. Cell Host & Microbe, 2020. **28**(6): p. 798-812.e6.
22. Olivier FAB *et al.*, The escape of *Candida albicans* from macrophages is enabled by the fungal toxin candidalysin and two host cell death pathways. Cell Reports, 2022. **40**(12).
23. Wellington M *et al.*, *Candida albicans* Triggers NLRP3-Mediated Pyroptosis in Macrophages. Eukaryotic Cell, 2014. **13**(2): p. 329.
24. Vylkova S, Lorenz Michael C, and Deepe George S, Phagosomal Neutralization by the Fungal Pathogen *Candida albicans* Induces Macrophage Pyroptosis. Infection and Immunity, 2017. **85**(2): p. e00832-16.
25. Moyes DL *et al.*, Candidalysin is a fungal peptide toxin critical for mucosal infection. Nature, 2016. **532**(7597): p. 64-68.
26. Mogavero S *et al.*, Candidalysin delivery to the invasion pocket is critical for host epithelial damage induced by *Candida albicans.* Cellular Microbiology, 2021. **23**(10): p. e13378.
27. Brunke S *et al.*, One Small Step for a Yeast - Microevolution within Macrophages Renders *Candida glabrata* Hypervirulent Due to a Single Point Mutation. PLOS Pathogens, 2014. **10**(10): p. e1004478.
28. Sprague JL *et al.*, *Candida albicans* translocation through the intestinal epithelial barrier is promoted by fungal zinc acquisition and limited by NFκB-mediated barrier protection. PLOS Pathogens, 2024. **20**(3): p. e1012031.
29. Deshpande A *et al.*, Reverse transcription progression and genome length regulate HIV-1 core elasticity and disassembly. PLOS Pathogens, 2025. **21**(6): p. e1013269.
30. Miyakawa K *et al.*, PHD3-VHL axis controls HIV-2 infection through oxygen-dependent hydroxylation and degradation of Vpx. PLOS Pathogens, 2025. **21**(6): p. e1013241.
31. Yin H *et al.*, Circovirus Rep evades immune restriction by disrupting cGAS oligomerization and phase separation. PLOS Pathogens, 2025. **21**(6): p. e1013244.
32. Pérez-Cantero A *et al.*, Heterochromatin de novo formation and maintenance in Plasmodium falciparum. PLOS Pathogens, 2025. **21**(6): p. e1013137.
33. Mojsiejczuk L *et al.*, Multiple introductions of equine influenza virus into the United Kingdom resulted in widespread outbreaks and lineage replacement. PLOS Pathogens, 2025. **21**(6): p. e1013227.
34. Hyodo A *et al.*, Evolutionary and structural basis of SLAMF1 utilization in morbilliviruses—Implications for host range and cross-species transmission. PLOS Pathogens, 2025. **21**(6): p. e1012990.
35. Kämmer P *et al.*, Survival Strategies of Pathogenic *Candida* Species in Human Blood Show Independent and Specific Adaptations. mBio, 2020. **11**(5): p. 10.1128/mbio.02435-20.
36. Last A *et al.*, *In vitro* infection models to study fungal–host interactions. FEMS Microbiology Reviews, 2021. **45**(5): p. fuab005.
37. Alonso-Roman R *et al.*, Organ-on-chip models for infectious disease research. Nature Microbiology, 2024. **9**(4): p. 891-904.
38. Maurer M *et al.*, A three-dimensional immunocompetent intestine-on-chip model as in vitro platform for functional and microbial interaction studies. Biomaterials, 2019. **220**: p. 119396.
39. Kaden T *et al.*, Modeling of intravenous caspofungin administration using an intestine-on-chip reveals altered *Candida albicans* microcolonies and pathogenicity. Biomaterials, 2024. **307**: p. 122525.
40. Fradin C *et al.*, Granulocytes govern the transcriptional response, morphology and proliferation of *Candida albicans* in human blood. Molecular Microbiology, 2005. **56**(2): p. 397-415.
41. Znaidi S *et al.*, Genomewide Location Analysis of *Candida albicans* Upc2p, a Regulator of Sterol Metabolism and Azole Drug Resistance. Eukaryotic Cell, 2008. **7**(5): p. 836-847.
42. Silver Peter M, Oliver Brian G, and White Theodore C, Role of *Candida albicans* Transcription Factor Upc2p in Drug Resistance and Sterol Metabolism. Eukaryotic Cell, 2004. **3**(6): p. 1391-1397.
43. Van Ende M, Wijnants S, and Van Dijck P, Sugar Sensing and Signaling in *Candida albicans* and *Candida glabrata.* Frontiers in Microbiology, 2019. **10**.
44. Naglik JR *et al.*, *Candida albicans* interactions with epithelial cells and mucosal immunity. Microbes and Infection, 2011. **13**(12): p. 963-976.

45. Lachat J *et al.*, Trans-cellular tunnels induced by the fungal pathogen *Candida albicans* facilitate invasion through successive epithelial cells without host damage. Nature Communications, 2022. **13**(1): p. 3781.
46. Wakade Rohan S, Wellington M, and Krysan Damian J, Temporal dynamics of *Candida albicans* morphogenesis and gene expression reveals distinctions between *in vitro* and *in vivo* filamentation. mSphere, 2024. **9**(4): p. e00110-24.
47. Brilhante RSN *et al.*, Exposure of *Candida parapsilosis* complex to agricultural azoles: An overview of the role of environmental determinants for the development of resistance. Science of The Total Environment, 2019. **650**: p. 1231-1238.
48. Chowdhary A *et al.*, Emergence of azole-resistant *Aspergillus fumigatus* strains due to agricultural azole use creates an increasing threat to human health. PLoS pathogens, 2013. **9**(10): p. e1003633-e1003633.
